# A robust vegetation index for remotely assessing chlorophyll content of dorsiventral leaves across several species in different seasons

**DOI:** 10.1186/s13007-018-0281-z

**Published:** 2018-02-14

**Authors:** Shan Lu, Fan Lu, Wenqiang You, Zheyi Wang, Yu Liu, Kenji Omasa

**Affiliations:** 1 School of Geographical Sciences, Northeast Normal University, 5268 Renmin Street, Changchun, 130024 China; 2 Graduate School of Agricultural and Life Sciences, The University of Tokyo, Yayoi 1-1-1, Bunkyo-ku, Tokyo, 113-8657 Japan

**Keywords:** Leaf chlorophyll content, Robust wavelength region, Adaxial, Abaxial, Reflectance

## Abstract

**Background:**

Leaf chlorophyll content (LCC) provides valuable information about plant physiology. Most of the published chlorophyll vegetation indices at the leaf level have been based on the spectral characteristics of the adaxial leaf surface, thus, they are not appropriate for estimating LCC when both the adaxial and abaxial leaf surfaces influence the spectral reflectance. We attempted to address this challenge by measuring the spectral reflectance of the adaxial and abaxial leaf surfaces of several plant species at different growth stages using a portable field spectroradiometer. The relationships between more than 30 published reflectance indices with LCC were analyzed to determine which index estimated LCC most effectively. Additionally, since the relationships determined on one set of samples might have poor predictive performances when applied to other samples, a robust wavelength region is required to render the spectral index generally applicable, regardless of the leaf surface or plant species.

**Results:**

The Modified Datt (MDATT) index, which is the ratio of reflectance difference defined as (R_λ3_ − R_λ1_)/(R_λ3_ − R_λ2_), exhibited the strongest correlation (R^2^ = 0.856, RMSE = 6.872 μg/cm^2^), with LCC of all the indices tested when all the leaf samples from the adaxial and abaxial surfaces were combined. The optimal wavelength regions, which were derived from the contour maps of R^2^ between the MDATT index and LCC for the datasets of one side or both leaf surfaces of each plant species and their intersection, indicated that the red-edge to near-infrared wavelength (723–885 nm) was optimal for λ_1_, while the red-edge region (697–771 nm) was optimal for λ_2_ and λ_3_. In these optimal wavelength regions, when the MDATT index was used to estimate LCC, an R^2^ higher than 0.8 could be obtained. The correlation of the MDATT index with LCC was the same when the positions of λ_2_ and λ_3_ were exchanged in the index.

**Conclusions:**

MDATT is proposed as an optimal index for the remote estimation of vegetation chlorophyll content across several plant species in different growth stages when reflectance from both leaf surfaces is considered. The red-edge to near-infrared wavelength (723–885 nm) for λ_1_, as well as the red-edge region (697–771 nm) for λ_2_ or λ_3_, are considered to be the most robust for constructing the MDATT index for estimating LCC, regardless of the leaf surface or plant species.

**Electronic supplementary material:**

The online version of this article (10.1186/s13007-018-0281-z) contains supplementary material, which is available to authorized users.

## Background

The detection of leaf chlorophyll content (LCC) is important for monitoring the physiological status of plants, assessing plant health, and estimating photosynthetic potential [1]. It is also helpful for understanding light acclimation mechanisms in higher plants [2], and furthermore, provides an indication of plant stress and senescence [3–6]. Although the traditional wet extraction analysis by field sampling provides the most accurate estimation of LCC status, such methods are not practical when estimates are required for large areas of vegetation. Non-destructive measurement of leaf spectral reflectance offers an alternative, instantaneous method for assessing the LCC of plants over large spatial scales.

Decades of research has gone into finding chlorophyll-sensitive wavelength regions to build vegetation indices using combinations of wavebands at different scales [1, 2, 5–7]. The normalized difference vegetation index (NDVI), based on the reflectance contrast between the red and the NIR (near infrared) [8], has been most commonly used for characterizing canopy LCC. However, remotely estimating canopy chlorophyll content via NDVI has been hindered by the shortcomings of broadband NDVIs derived from red wavebands positioned in the chlorophyll absorption pit (at approximately 670–680 nm) and bands positioned in the NIR plateau (between 750 and 900 nm) [9–12]. Furthermore, broadband NDVIs are only effective in distinguishing broad differences in vegetation conditions [9, 13] but not effective in assessing detailed canopy LCC due to their saturation at a high leaf area index (LAI).

Studies based on narrowband spectra have been conducted to develop the vegetation indices, which take the form of simple ratios (SR), simple difference (SD), normalized difference (ND), as well as other forms to estimate LCC [14–23]. The vegetation indices are initially developed at the leaf level using hyperspectral data because an assessment of the practicality of such a method at leaf level is regarded as a first step for further research at the canopy scale and for the remote estimation of LCC from satellite observation, which is essential in ecosystem modeling [24, 25].

However, most of these vegetation indices based on narrowband spectra at the leaf level were developed by correlating the LCC and reflectance derived only from the adaxial surface. The leaves of some plants, such as *Populus*, are easily agitated by light wind, causing the leaves to move from side to side. The leaves also curl and expose their abaxial surface in extreme drought conditions. These factors may result in the remote sensor obtaining the reflectance information not only from the adaxial surface but also the abaxial surface. It is therefore potentially unsuitable to remotely assess LCC by measuring only the adaxial surface reflectance. The differences in the optical properties of bifacial leaves have been well documented [26, 27]. To the best of our knowledge, very few studies have focused on the effects of bifacial structures on the accuracy of the remote estimation of LCC. Lu et al. [28] assessed LCC remotely, accounting for the dorsiventral structure of the leaves of two woody plants, namely white poplar and Chinese elm. Although the bifacial structure-insensitive index of Modified Datt (MDATT) was developed in our previous study, it is not clear whether this index is universally applicable to a wide range of species and leaf structures. Thus, the effectiveness of the index across a broader range of plant species requires investigation.

Generally, different wavelength combinations are selected for a vegetation index with a specific formula depending on the different samples analyzed by statistical methods. For example, some research utilized reflectance at 420, 550, 605, 695, 700, 710, or 750 nm for the ratio index [29]. It is also common to use other indices with different formulas. However, the rationale for the selection of the wavelengths has seldom been discussed. This is not practical for researchers who wish to apply an index in their study based on the selection of a particular wavelength combination. Thus, a set of optimal wavelength regions may be more appropriate than specific wavelengths when reasonable estimation accuracy is required, whether the dataset is derived from the adaxial surface, abaxial surface, both surfaces, or different plant species with various leaf structures. The development of vegetation indices at either the leaf or canopy level often focuses on mitigating unwanted reflectance effects, while increasing the indices’ sensitivity to those biochemical and biophysical parameters of vegetation [9]. Thus, it is important to reduce the effect of leaf structures on the reflectance to accurately correlate the vegetation indices with LCC. The vegetation indices for which the effect of leaf structure has been reduced at the leaf level could be scaled up to the canopy level with fewer issues.

One of the aims of the present study is to examine which index associated with a specific formula is the most effective for estimating LCC across various plant species with different structural features when adaxial, abaxial, or bifacial reflectance is considered. Additionally, leaves collected from different growth stages are also considered to render the chosen index applicable for LCC estimation across multiple seasons. The possible factors that influence the selection of various feasible wavelengths for different plant species are discussed. Another aim is to identify optimal wavelength regions for the best performing vegetation index to estimate LCC regardless of whether the reflectance data were collected from only one or both leaf surfaces as well as different plant species.

## Methods

Developing, fully expanded, and senescent leaves of white poplar (*Populus alba*) (n = 54), narrow-leaved oleaster (*Elaeagnus angustifolia* L.) (n = 56), Manchurian lilac (*Syringa reticulata* (Blume) H. Hara var. *amurensis* (Rupr.) J. S. Pringle) (n = 63), Chinese elm (*Ulmus pumila var. pendula*) (n = 67), Virginia creeper (*Parthenocissus quinquefolia*) (n = 57), grapevine (*Vitis* L.) (n = 60), and torch tree (*Rhus typhina*) (n = 56) were collected from the campus of the Northeast Normal University, Changchun, China, in the spring, summer, and autumn of 2015. Leaves that were homogeneous in color as well as those with visible symptoms of damage were used in the experiments to account for possible variation in chlorophyll content in these types of leaves.

The leaf samples were first transported from the field to the laboratory, and then the adaxial and abaxial leaf surface reflectance spectra were measured in the spectral range of 400–1000 nm at a spectral resolution of 1.4 nm with a portable hand-held spectrometer (FieldSpec^®^ HandHeld 2, Analytical Spectral Devices, Boulder, CO, USA). The reflectance measurements were performed using a leaf clipper equipped with an internal halogen source directly attached to the leaf surface. A Spectralon^®^ diffuse reflectance standard (Labsphere, North Sutton, NH, USA) was scanned before each new sample. The reflectance of the sample was calculated as the ratio of leaf radiance divided by the reflectance standard radiance at wavelength λ; the average of three separate scans from each sample was recorded. The reflectance spectra were transformed to the published indices that have been recommended as excellent indicators of foliar chlorophyll.

LCC was determined from the same leaf samples used for the reflectance measurements. Circular disks with a diameter of 6 mm were punched from the leaves using a cork borer and extracted with 95% ethanol using a mortar and pestle. The pigment extracts were centrifuged for 3–5 min in glass tubes to render the extract fully transparent. The resulting extracts were immediately assayed spectrophotometrically with a Lambda 900 spectrophotometer (Perkin-Elmer, Waltham, MA, USA). Specific absorption coefficients of Chl a and Chl b reported by Wintermans and De Mots [30] were used to calculate the chlorophyll content (μg/cm^2^).

More than 30 published chlorophyll indices (Table 1) were derived from the reflectance datasets. The selected spectral indices were considered to be good candidates for the estimation of plant LCC. In addition, we calculated two-band and three-band indices, i.e. SD, SR, ND, and MDATT, using the wavebands (λ_1_, λ_2_, and λ_3_) in the 400–1000 nm region to select the best wavelength combination for assessment of LCC, as shown in Eqs. (1–4). These indices were evaluated using a custom developed computer program to traverse each wavelength combination for the indices. The optimal combination that exhibited high correlation to the biochemically measured LCC was then selected.

**Table 1:** The existing vegetation indices used in this study

Vegetation index	Formula	Reference
1/*R*_700_	1/*R*_700_	[31]
*R*_680_	*R*_680_	[18]
1/*R*_700_ − 1/*R*_750_	1/*R*_700_ − 1/*R*_750_	[1]
1/*R*_550_ − 1/*R*_750_	1/*R*_550_ − 1/*R*_750_	[1]
SD	*R*_λ1_ − *R*_λ2_	This paper
*R*_750_/*R*_550_	*R*_750_/*R*_550_	[32]
*R*_750_/*R*_700_	*R*_750_/*R*_700_	[32]
*R*_860_/*R*_550_	*R*_860_/*R*_550_	[33]
*R*_672_/*R*_550_	*R*_672_/*R*_550_	[33]
PSSR_*a*_	*R*_800_/*R*_680_	[17]
PSSR_*b*_	*R*_800_/*R*_635_	[17]
*R*_800_/*R*_650_	*R*_800_/*R*_650_	[18]
*R*_800_/*R*_675_	*R*_800_/*R*_675_	[18]
*R*_450_/*R*_550_	*R*_450_/*R*_550_	[34]
*R*_750_/*R*_710_	*R*_750_/*R*_710_	[35]
*R*_950_/*R*_680_	*R*_950_/*R*_680_	[36]
SR	*R*_λ1_/*R*_λ2_	This paper
NDI	(*R*_750_ − R_705_)/(*R*_750_ + *R*_705_)	[37]
PSND_*b*_	(*R*_800_ − *R*_635_)/(*R*_800_ + *R*_635_)	[17]
(*R*_800_ − *R*_650_)/(*R*_800_ + *R*_650_)	(*R*_800_ − *R*_650_)/(*R*_800_ + *R*_650_)	[18]
(*R*_800_ − *R*_675_)/(*R*_800_ + *R*_675_)	(*R*_800_ − *R*_675_)/(*R*_800_ + *R*_675_)	[18]
ND	|*R*_λ1_ − *R*_λ2_|/(*R*_λ1_ + *R*_λ2_)	This paper
*D*_754_/*D*_704_	*D*_754_/*D*_704_	[38]
*RII*	$\int_{705}^{750} {({{R_{\lambda } } \mathord{\left/ {\vphantom {{R_{\lambda } } {R_{705} - 1}}} \right. \kern-0pt} {R_{705} - 1}})} d\lambda$	[39]
*D*_730_	*D*_730_	[39]
*D*_710_	*D*_710_	[40]
*D*_740_	*D*_740_	[40]
VOG_2_	(*R*_734_ − *R*_747_)/(*R*_715_ + *R*_726_)	[23]
CARI	(|(a*670 + *R*_670_ + b)|/(a^2^ + 1)^0.5^)*(*R*_700_/*R*_670_) [a = (*R*_700_ − *R*_550_)/150; b = *R*_550_ − (a*550)]	[41]
*R*_672_/(*R*_550_ × *R*_708_)	*R*_672_/(*R*_550_ × *R*_708_)	[33]
*R*_860_/(*R*_550_ × *R*_708_)	*R*_860_/(*R*_550_ × *R*_708_)	[33]
MCARI	[(*R*_700_ − *R*_670_) − 0.2*(*R*_700_ − *R*_550_)]*(*R*_700_/*R*_670_)	[42]
TCARI/OSAVI	3*[(*R*_700_ − *R*_670_) − 0.2*(*R*_700_ − *R*_550_)*(*R*_700_/*R*_670_)]/[(1 + 0.16)*(*R*_800_–*R*_670_)/(*R*_800_ + *R*_670_ + 0.16)]	[43]
TCARI	3*[(*R*_700_ − *R*_670_) − 0.2*(*R*_700_ − *R*_550_)*(*R*_700_/*R*_670_)]	[44]
*R*_705_/(*R*_717_ + *R*_491_)	*R*_705_/(*R*_717_ + *R*_491_)	[45]
*R*_434_/(*R*_496_ + *R*_401_)	*R*_434_/(*R*_496_ + *R*_401_)	[45]
(*R*_850_ − *R*_710_)/(*R*_850_ − *R*_680_)	(*R*_850_ − *R*_710_)/(*R*_850_ − *R*_680_)	[22]
(*R*_719_ − *R*_726_)*/*(*R*_719_ − *R*_743_)	(*R*_719_ − *R*_726_)*/*(*R*_719_ − *R*_743_)	[28]
MDATT index: (*R*_λ3_ − *R*_λ1_)/(*R*_λ3_ − *R*_λ2_)	MDATT index: (*R*_λ3_ − *R*_λ1_)/(*R*_λ3_ − *R*_λ2_)	This paper

1 $$SD(R_{{\lambda_{1} }} ,R_{{\lambda_{2} }} ) = R_{{\lambda_{1} }} - R_{{\lambda_{2} }}$$

2 $$SR(R_{{\lambda_{1} }} ,R_{{\lambda_{2} }} ) = \frac{{R_{{\lambda_{1} }} }}{{R_{{\lambda_{2} }} }}$$

3 $$ND(R_{{\lambda_{1} }} ,R_{{\lambda_{2} }} ) = {{\left| {R_{{\lambda_{1} }} - R_{{\lambda_{2} }} } \right|} \mathord{\left/ {\vphantom {{\left| {R_{{\lambda_{1} }} - R_{{\lambda_{2} }} } \right|} {\left( {R_{{\lambda_{1} }} + R_{{\lambda_{2} }} } \right)}}} \right. \kern-0pt} {\left( {R_{{\lambda_{1} }} + R_{{\lambda_{2} }} } \right)}}$$

4 $${\text{M}}DATT = {{\left( {R_{{\lambda_{3} }} - R_{{\lambda_{1} }} } \right)} \mathord{\left/ {\vphantom {{\left( {R_{{\lambda_{3} }} - R_{{\lambda_{1} }} } \right)} {\left( {R_{{\lambda_{3} }} - R_{{\lambda_{2} }} } \right)}}} \right. \kern-0pt} {\left( {R_{{\lambda_{3} }} - R_{{\lambda_{2} }} } \right)}}$$

In order to obtain the optimal wavelength regions for the indices, the R^2^ values obtained from the correlation analysis between the spectral indices with all combinations of spectral wavelengths and LCC were sorted from lowest to highest. Next, the contour maps of R^2^ between chlorophyll content and the vegetation indices with two wavelengths on the x- and y-axes were plotted. For the MDATT indices, contour maps for each two-wavelength combination were plotted to assess the statistical significance of the spectral indices for all combinations. The contour R^2^ maps for each dataset, including the single or double leaf surfaces for each plant species, were intersected to obtain robust wavelength regions for the spectral indices.

## Results

### Spectral reflectance differences between the adaxial and abaxial leaf surfaces

**Fig. 1:**
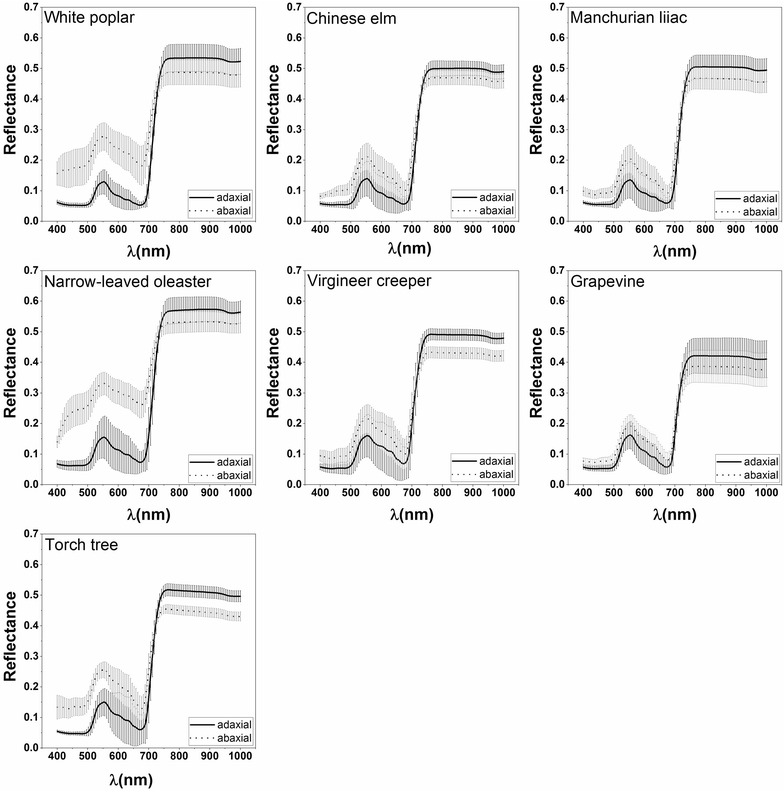
The reflectance of the adaxial and abaxial leaf surfaces for each plant species

**Fig. 2:**
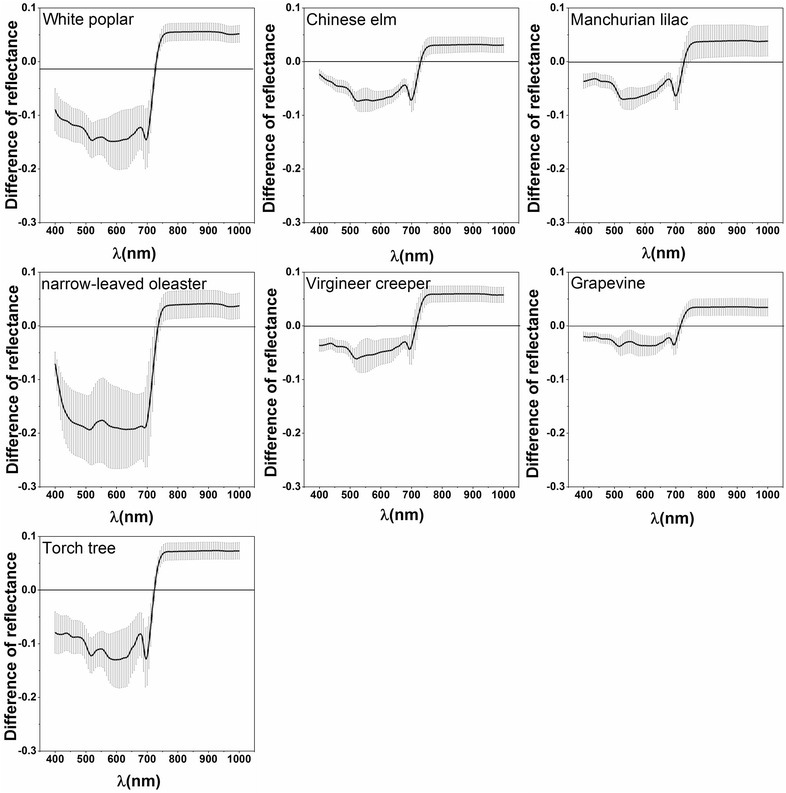
The spectral differences between the adaxial and abaxial leaf surfaces for each plant species

The reflectance spectra investigated for the adaxial and abaxial leaf surfaces are shown in Fig. 1. It is evident that the reflectance was much lower in the visible wavelengths (400–700 nm) for the adaxial surface than for the abaxial surface in all species. On the contrary, the reflectance of the adaxial surface was higher than that of the abaxial surface in the near-infrared wavelengths (700–1000 nm). In addition, distinct differences in the spectral reflectance of the adaxial and abaxial surfaces of the different plants were observed. For example, the reflectance differences of white poplar and narrow-leaved oleaster were significantly larger than for the other species tested (Fig. 1) in the visible wavelengths. The differences in reflectance between the adaxial and abaxial surfaces are shown in Fig. 2. There was a clear difference between the upper and lower surfaces in white poplar and narrow-leaved oleaster as their abaxial surfaces are covered in dense hair, but less variation was found in the leaves of the other species. The least reflectance differences between the adaxial and abaxial surfaces occurred almost entirely in the 716–732 nm wavelength range (Fig. 2).

**Fig. 3:**
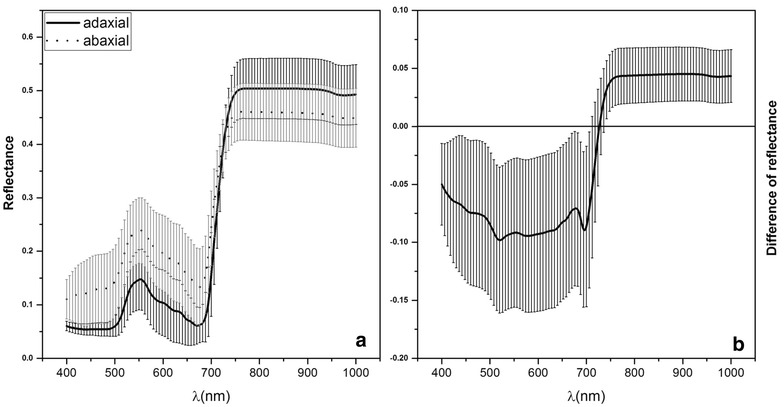
The mean spectral reflectance on the adaxial and abaxial surface (**a**) and reflectance differences between the two leaf surfaces (**b**)

The spectral reflectance values and the average differences between all the samples are shown in Fig. 3. A similar contrasting relationship as that observed in the bifacial reflectance for each plant leaf sample was observed. The reflectance on the abaxial leaf surface was higher in the visible wavelength and lower in the near-infrared wavelength (Fig. 3a). The standard deviation of the spectral difference (Fig. 3b) between the two leaf surfaces was larger than that of individual plant samples, but the lowest reflectance difference was 727 nm, which was still at the red-edge range mentioned above. Meanwhile, almost all the differences in reflectance were significant based on a paired *t* test (*p* < 0.01), except for the 718–725 nm wavelength range.

### Relationship between LCC and the MDATT indices derived from each plant

**Fig. 4:**
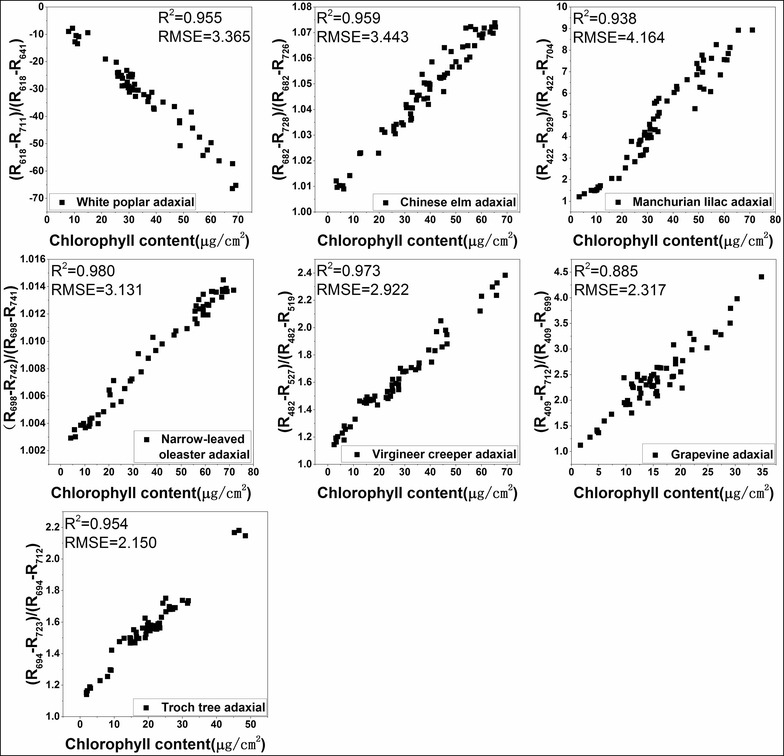
The relationship between the best performing MDATT and LCC for each plant species on the adaxial surface

**Fig. 5:**
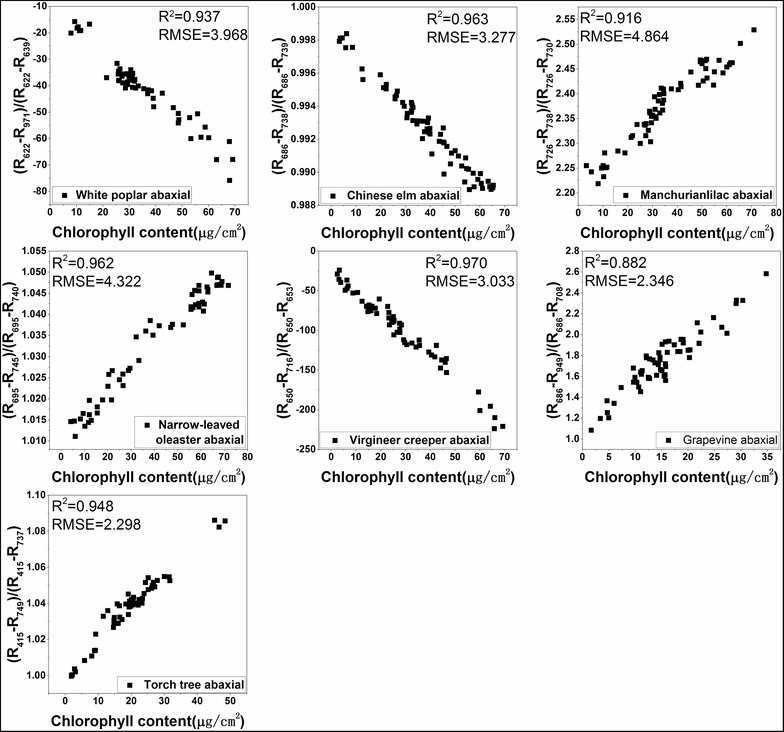
The relationship between the best performing MDATT and LCC for each plant species on the abaxial surface

**Fig. 6:**
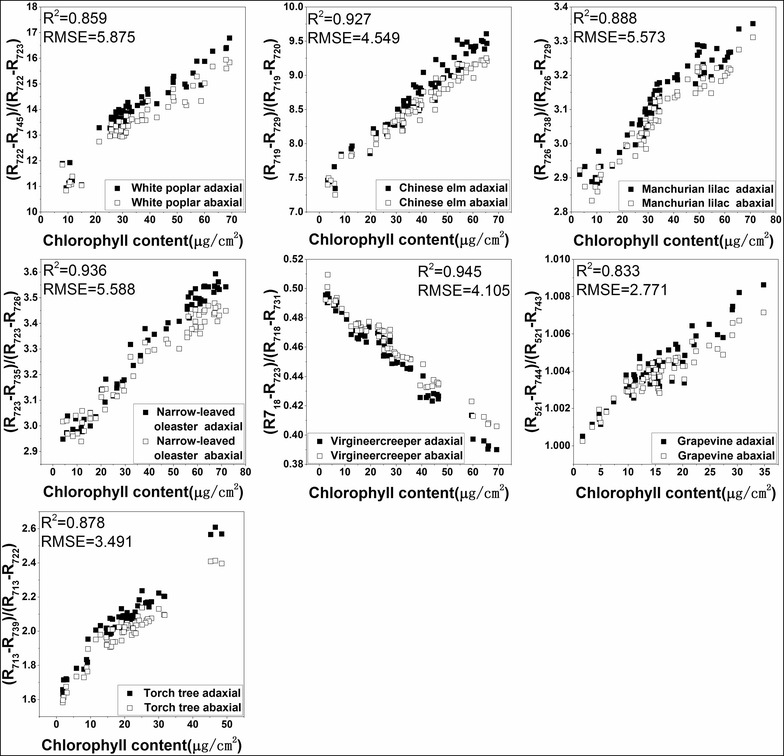
The relationship between the best performing MDATT and LCC for each plant species when the adaxial and abaxial surfaces data are combined

The R^2^ values between LCC and all the spectral indices for each plant are shown in Additional file 1: Table S1. MDATT exhibited the best correlation with LCC for most of the adaxial, abaxial, and mixed datasets of each plant species, with only a few exceptions. The exceptions occurred on the abaxial surface dataset of the narrow-leaved oleaster, Manchurian lilac, and grapevine. Although MDATT did not have the highest correlation with LCC for this dataset, the R^2^ was not much lower than the best performing indices. For example, in the abaxial dataset of the narrow-leaved oleaster, the highest R^2^ provided by the SR index was 0.981, but MDATT showed an R^2^ value of 0.980 for the same dataset. The largest R^2^ gap between MDATT and the best performing vegetation index was only 0.007. The small R^2^ difference between MDATT and the well-performing vegetation indices indicated that MDATT correlated well with LCC despite it not always being ranked first among the indices tested. The scatter plots of the MDATT index and LCC for the adaxial, abaxial, and mixed datasets are shown in Figs. 4, 5 and 6. It is clear that MDATT was highly correlated with LCC for almost all datasets. With regards to the specific wavelengths selected for the individual datasets, most of the best wavelengths were at the red-edge except for the adaxial dataset of Manchurian lilac, Virginia creeper, and grapevine, the abaxial dataset of torch tree, and the adaxial and abaxial dataset of grapevine.

### Relationship between LCC and the MDATT indices derived from all plant samples

**Fig. 7:**
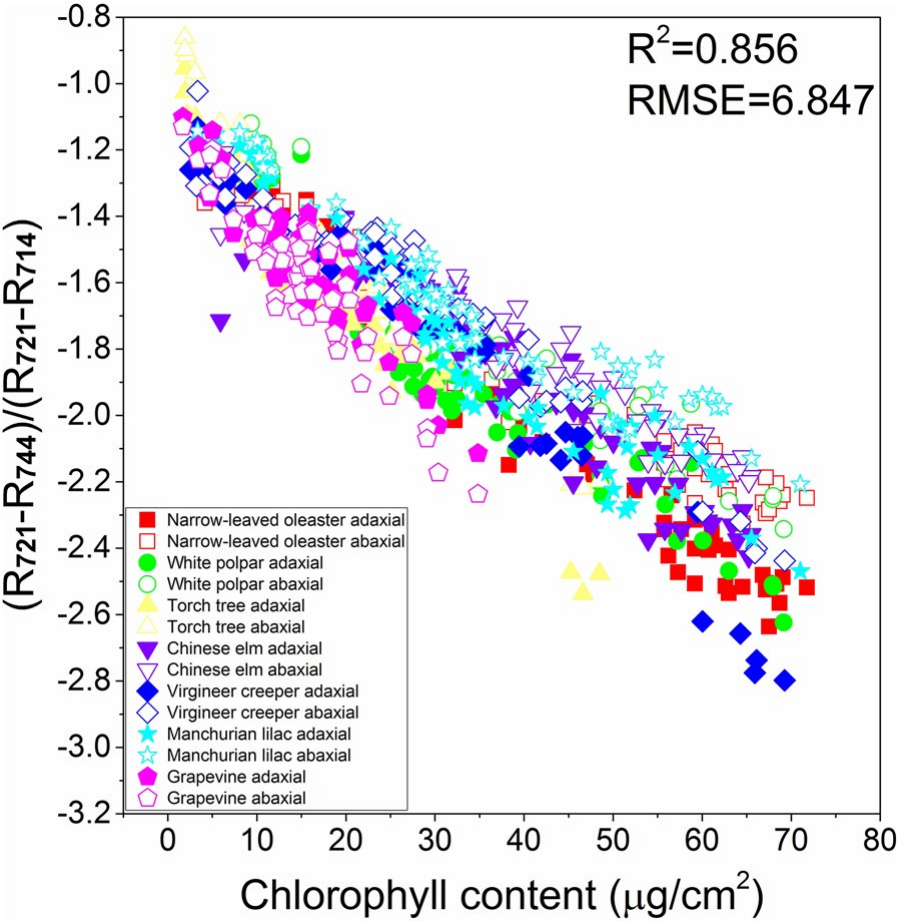
The relationship between the best performing MDATT and LCC for all the plant species on both adaxial and abaxial surfaces

The R^2^ and RMSE (root mean standard error) values between LCC and the spectral indices for all the plant samples are shown in Table 2. The vegetation index most strongly correlated with LCC was MDATT regardless of whether the adaxial, abaxial, or mixed surfaces dataset was used in the regression analysis. In particular, MDATT performed best for the data when all the leaf samples were combined. It is of significance that MDATT could predict the LCC not only across several species, including woody and liana plants, but also plants with different leaf surface structures. Although our previous study [28] elaborated on the significance of MDATT in estimating LCC for the different leaf surfaces of two deciduous plants, namely white poplar and Chinese elm, it was not previously shown that MDATT also exhibits good predictability for more plant species when both the adaxial and abaxial leaf surface information is considered. The relationship between the best performing MDATT of (R_721_ − R_744_)/(R_721_ − R_714_) and LCC is shown in Fig. 7. They exhibited a good linear relationship with an R^2^ of 0.856 and RMSE of 6.847 μg/cm^2^.

**Table 2:** The coefficients of determination and RMSE of the vegetation indices for estimating the LCC on adaxial, abaxial, and both surfaces (only the top 15 vegetation indices with high R^2^ value were listed)

All plants								
Adaxial and abaxial			Adaxial			Abaxial		
VI	R^2^	RMSE (μg/cm^2^)	VI	R^2^	RMSE (μg/cm^2^)	VI	R^2^	RMSE (μg/cm^2^)
MDATT: (R_721_ − R_744_)/(R_721_ − R_714_)	0.856	6.872	MDATT: (R_691_ − R_745_)/(R_691_ − R_736_)	0.910	5.426	MDATT: (R_688_ − R_745_)/(R_688_ − R_736_)	0.912	5.370
(R_719_ − R_726_)/(R_719_ − R_743_)	0.801	8.040	SR:R_859_/R_721_	0.907	5.514	D_754_/D_704_	0.854	6.904
D_754_/D_704_	0.699	9.907	ND: (R_742_ − R_741_)/(R_742_ + R_741_)	0.906	5.545	(R_850_ − R_710_)/(R_850_ − R_680_)	0.790	8.289
(R_850_ − R_710_)/(R_850_ − R_680_)	0.690	10.055	VOG2:(R_734_ − R7_47_)/(R_715_ + R_726_)	0.905	5.559	(R_719_ − R_726_)/(R_719_ − R_743_)	0.788	8.310
SR: R_742_/R_760_	0.615	11.199	R_750_/R_710_	0.894	5.878	SD: R_741_ − R_748_	0.787	8.559
ND: (R_745_ − R_751_)/(R_745_ + R_751_)	0.614	11.205	SD: R7_45_ − R_744_	0.889	6.026	D_740_	0.771	10.105
SD: R_704_ − R_680_	0.615	11.254	D_740_	0.880	6.270	SR: R_740_/R_759_	0.690	10.106
D_740_	0.580	11.698	RII	0.873	6.435	ND: (R_740_ − R_760_)/(R_740_ + R_760_)	0.690	10.267
VOG2	0.548	12.127	R_750_/R_700_	0.858	6.799	D_730_	0.677	10.845
TCARI	0.537	12.279	D_754_/D_704_	0.848	7.053	VOG2	0.640	11.807
D_730_	0.520	12.507	(R_719_ − R_726_)/(R_719_ − R_743_)	0.845	7.110	TCARI	0.521	12.505
MCARI	0.445	13.444	D_730_	0.830	7.456	TCARI/OSAVI	0.487	12.945
R_750_/R_710_	0.437	13.536	1/R_700_ − 1/R_750_	0.820	7.674	R_750_/R_710_	0.447	13.442
TCARI/OSAVI	0.432	13.605	(R_850_ − R_710_)/(R_850_ − R_680_)	0.801	8.066	RII	0.413	13.842
R_672_/(R_550_*R_708_)	0.420	13.748	NDI	0.800	8.081	MCARI	0.392	14.084

The wavelengths used to develop MDATT for all the plant samples fell within the scope of the red-edge irrespective of the use of a single surface dataset or mixed surface dataset (Table 2). Although the wavelengths selected in this study differed slightly from the results of Lu et al. [28], the wavelength range was very similar to the above study and also exhibited zero reflectance difference between the adaxial and abaxial leaf surfaces.

### Robust wavelength regions for estimating LCC from MDATT

**Fig. 8:**
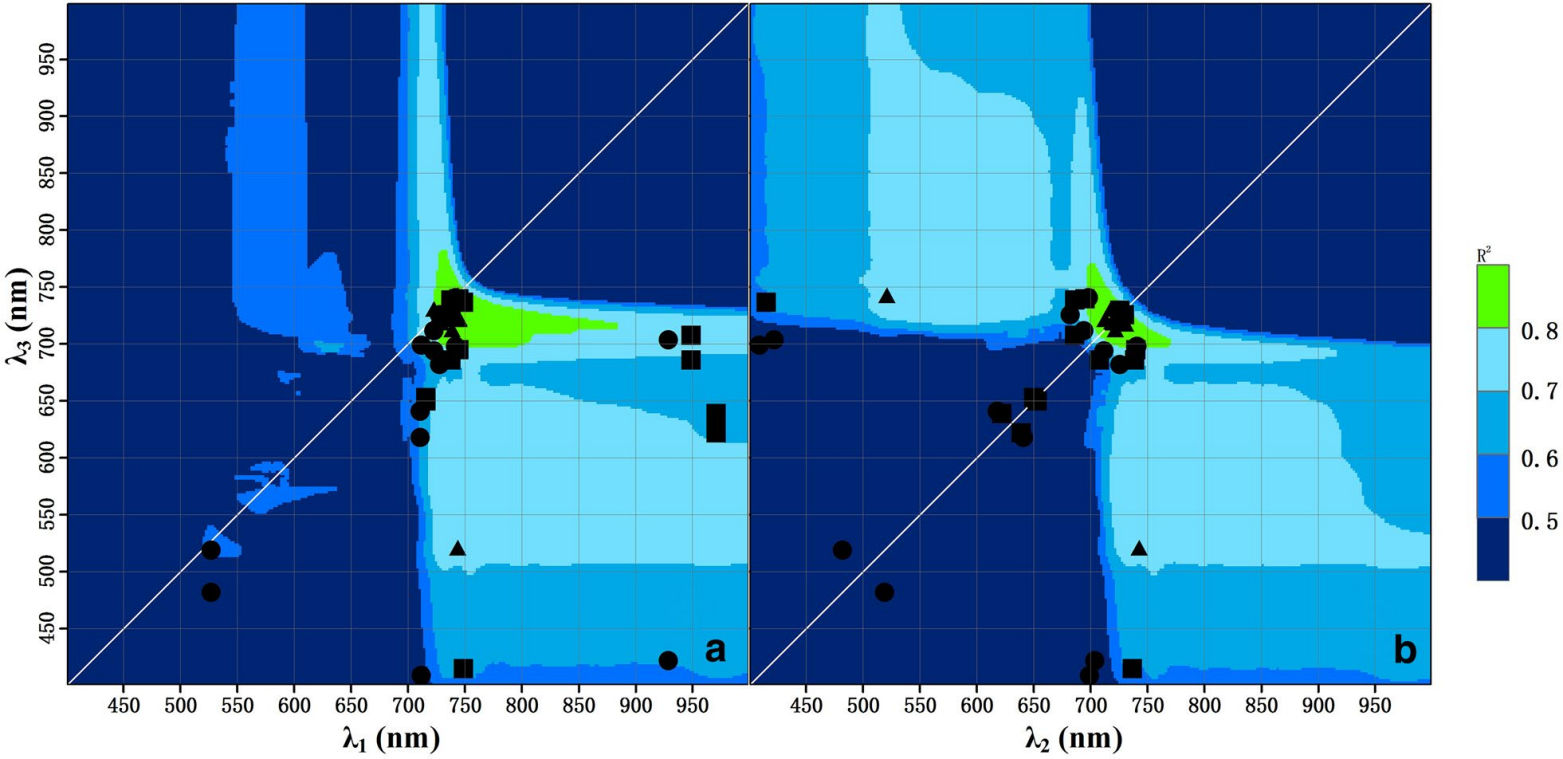
Intersected R^2^ contour map of the adaxial, abaxial, and both leaf surfaces for each plant species (the dots represent the wavelength combination with the highest R^2^ for the adaxial surface of each plant dataset, the squares represent the wavelength combination with the highest R^2^ for the adaxial surface of each plant dataset, and the triangles represent the wavelength combination with the highest R^2^ combination for both surfaces of each plant dataset)

**Fig. 9:**
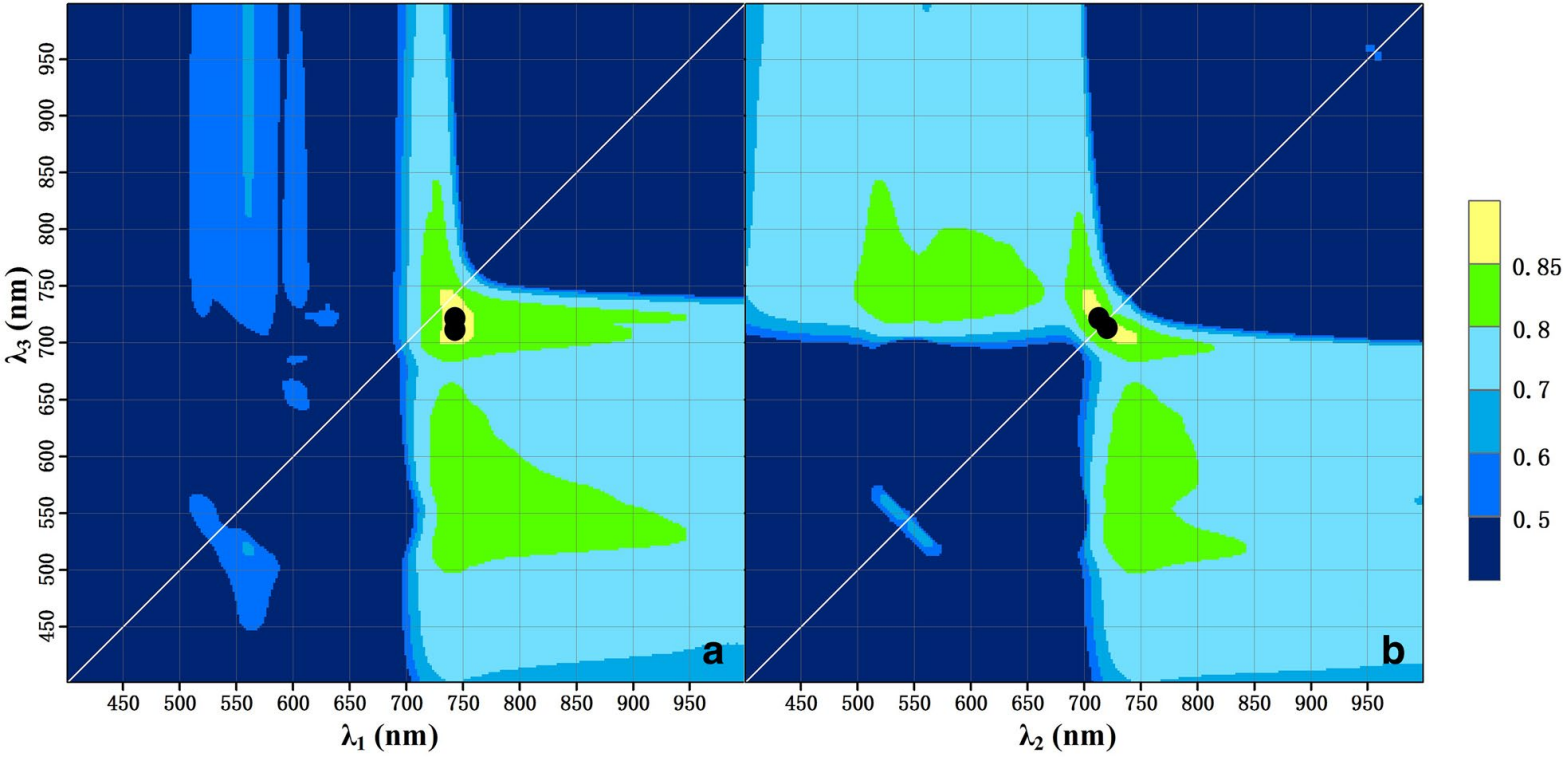
Contour maps for R^2^ between LCC and the MDATT index with the λ_1_ and λ_3_ (**a**), and λ_2_ and λ_3_ (**b**) combinations derived from the adaxial and abaxial leaf reflectance for all the plants

The contour maps of R^2^ provided efficient extraction of significant wavelengths as well as sufficient extent of the effective regions for the estimation of LCC. Interestingly, the contour maps of R^2^ for the combination of λ_2_ and λ_3_ were completely symmetrical. This indicated that these two wavelengths could be replaced by one another in the MDATT index. Although the replacement may result in a different MDATT index value, the correlation with LCC would remain unchanged. Thus, the contour maps for λ_1_ and λ_3_ as well as λ_1_ and λ_2_ were the same and only the contour maps of R^2^ for the λ_1_ and λ_3_ as well as λ_2_ and λ_3_ combinations are shown in Additional file 2: Figure S1, Additional file 3: Figure S2, Additional file 4: Figure S3, Additional file 5: Figure S4, Additional file 6: Figure S5, Additional file 7: Figure S6 and Figs. 8 and 9.

The contour maps for each leaf surface of the individual plant species showed a variety of sensitive regions (Additional file 2: Figure S1, Additional file 3: Figure S2, Additional file 4: Figure S3, Additional file 5: Figure S4). For example, the sensitive region (R^2^ > 0.9) for the adaxial or abaxial surface of most of the species was very broad for almost the entire near-infrared spectrum (700–1000 nm). However, when the data from both leaf surfaces were combined, the sensitive regions for most of the plant species shrank to a few distinct regions such as the red-edge wavelength and (or) green wavelengths (Additional file 6: Figure S5, Additional file 7: Figure S6). The R^2^ decreased to even lower than 0.85. The intersected contour map (Fig. 8) derived from the adaxial, abaxial, and both leaf surface maps for each plant species (21 in total) showed that the most robust wavelength combination was located at the red-edge area (697–771 nm) for λ_2_ and λ_3_, but was broader for λ_1_ and extended to the near-infrared (723–885 nm). Within this region, the R^2^ between MDATT and LCC was higher than 0.8 for any arbitrary dataset regardless of whether the reflectance was measured from one or both leaf surfaces, or even different plant species. In the case of the samples from a combination of all plant species, the sensitive wavelength region was reduced to only the red-edge (730–760 nm for λ_1_; 699–747 nm for λ_2_ and λ_3_) for the MDATT index without any other significant regions, with an R^2^ higher than 0.85 (Fig. 9).

## Discussion

The reflectance from the adaxial and abaxial surfaces differed distinctly, and the structural differences between the leaf surfaces are thought to account for this. When radiant energy strikes a leaf, part of it is reflected by the leaf surface, and the rest enters the leaf, where it is scattered by the mesophyll structure. Part of the internally scattered radiation is reflected back out of the surface of incidence, and the remainder is transmitted through the leaf [46]. The internally scattered radiation is also absorbed at specific wavelengths by various leaf biochemicals [22]. Because both the leaf surface and the internal mesophyll structures of the adaxial and abaxial leaf blades are entirely different, the spectral reflectance for the same leaf blade is different. The compact structure of the palisade layer was found to facilitate penetration of adaxial light into the spongy tissue [47], where the light is scattered due to the large number of cell-air interfaces of the more loosely packed cells. For abaxial illumination, the scattering by the spongy mesophyll cells occurs before light can be guided into the leaf interior, which increases the reflectance [48]. The marked differences in leaf surface structures also further reinforce the reflectance differences. For example, the abaxial surface of the leaves of white poplar and narrow-leaved oleaster is covered with dense hair, which results in a large difference between the adaxial and abaxial surface structures. The difference between the reflectance obtained from the adaxial and abaxial leaf surfaces for these two species differed more remarkably than for the other plant species in this study.

The spectral indices developed thus far have performed well in estimating LCC using only adaxial leaf surface spectral information [14–23], but the majority of indices are compromised when the adaxial and abaxial surface reflectance exists at the same time as a result of the different structures of the two leaf surfaces. The key factors for determining an optimal spectral index are the formula and the wavelengths applied in the formula. The formula should function to remove the physical effects of leaf structures or surface roughness on the reflectance. Furthermore, the selected wavelengths should be sensitive to the chemicals tested. MDATT is a spectral index that removes the surface and internal structural effects of the leaf surface on the reflectance and then derives an analytical relationship between leaf reflectance and biochemical content such as LCC [28]. By sampling the leaves of several plant species, including both woody and liana plants, MDATT was demonstrated to be optimal not only for estimating LCC using reflectance for cases where the incident radiant strikes the adaxial leaf surface but also from the abaxial surface or a combination thereof.

Because the MDATT formula removes the effect of the outer surface and internal structures of the different leaf surfaces, the wavelength regions selected for effective MDATT are determined by biochemicals, such as pigments. However, some exceptions exist for the selection of the best wavelength regions beyond the red-edge; for example, the adaxial and abaxial mixed dataset of grapevine, in which 521–526 nm was chosen for λ_3_ of MDATT. Grapevine is unusual with respect to the other plants tested in this study because the leaves may contain other pigments such as anthocyanins, and these may be at different concentrations since the leaf samples were taken from almost every stage of plant growth. The color of anthocyanins in the tissue changes with the environment; for example, in response to pH. Anthocyanin color was found to change from red to dark brown when the pH varied from 3.0 to 8.0 [49], and it controls the leaf reflectance, particularly in the visible wavelengths. The influence of anthocyanins on the grapevine dataset may explain the best wavelength region near 520 nm for λ_3_. Nevertheless, applying the red-edge region in MDATT also showed a good relationship with LCC for this dataset. Thus, the best wavelength region near 520 nm for this dataset appeared only by chance, while the red-edge region was more favorable for almost all the datasets.

In addition, very short wavelength regions were also found to be good for the adaxial dataset of Manchurian lilac and Virginia creeper and the abaxial dataset of torch tree. The effects of sampling stage, relative contents of different pigments in the leaves, and the pH level perhaps resulted in the variation of the best wavelength for MDATT. However, in contrast to other regions, the red-edge region always appeared frequently.

The Datt index is defined as a ratio of the differences in reflectance at three wavelengths: 850, 710, and 680 nm [21]. The reflectance at 850 nm is the minuend of the numerator and denominator, and the reflectances at 710 and 680 nm are the subtrahend of the numerator and denominator, respectively. The 710 nm wavelength was used because it is the most sensitive to chlorophyll content, 680 nm was used due to its strong chlorophyll absorption, and 850 nm was selected as a result of maximum scattering in the near-infrared wavelengths. However, why these wavelengths were utilized at the fixed position in the formula of the index was not determined. The results of this study revealed that it is not always certain that the visible wavelengths are required in the MDATT index to estimate the LCC when the reflection of individual and both surfaces from a variety of plant species is considered. The exchangeable λ_2_ and λ_3_ also showed that the position of each wavelength is not always fixed in the MDATT index.

Furthermore, most of the observed relationships between MDATT and LCC for single leaf surface reflectances were better than for mixed surfaces. It was assumed that the reflectance difference between the adaxial and abaxial leaf resulted in a worse correlation of the spectral index with LCC when the reflectance datasets of the two leaf surfaces were combined. Thus, although MDATT could remove the structural effect on reflectance between the adaxial and abaxial leaf surfaces compared with the other spectral indices, the structural effects remained partly due to the fact that MDATT was based on a semi-empirical reflectance model by Baret et al. [50].

The intersected contour map of R^2^ (Fig. 8) derived from the adaxial, abaxial, and both leaf surfaces of each plant species provided a robust wavelength region for each band applied in the MDATT index. The red-edge region and the near-infrared (723–885 nm) for λ_1_ and 697–771 nm for λ_2_ and λ_3_ were considered to be optimal because any combination of this region would obtain a reasonable correlation (R^2^ > 0.8) with LCC. Thus, the robust wavelength region, but not any specific best wavelength combination, is a good choice for use in MDATT in order to obtain a reasonable accuracy for LCC estimation considering adaxial, abaxial, both leaf surfaces, or different plant species. Spectral indices based on reflectance in the red-edge region constitute better indicators for LCC than other regions, as documented in many studies [1, 37, 51], and can be explained by the fact that red-edge reflectance is considered to be most closely associated with chlorophyll, with very little influence from other pigments. Furthermore, the red-edge region was optimal for the adaxial, abaxial, and mixed surface datasets of each plant species. Additionally, the smallest reflectance differences occurred on the red-edge spectral region.

It should be noted that the use of robust wavelength regions for each band involved in the MDATT index is very necessary to make the vegetation index more available. Different wavelength combinations may be derived as the best performed index for specific leaf samples from different plant species or growth stages. For example, the MDATT index in our previous study to estimate LCC was (R_719_ − R_726_)/(R_719_ − R_743_) for data from both leaf surfaces of white poplar and Chinese elm. However, when the leaf samples were taken from more plant species that experience seasonal changes in their leaves, such as in this study, the most effective MDATT index was (R_721_ − R_744_)/(R_721_ − R_714_) with a small wavelength shift from the previous one. However, both of them fell into the robust wavelength regions found in this study and gave reasonable accuracies as shown in Additional file 1: Table S1 and Table 2. Thus, the robust wavelength regions could provide an optimal MDATT index, although it may not always be the best MDATT index.

Incidentally, the NDVI may not be suitable for the case in which the adaxial and abaxial leaf surfaces are considered because the formula cannot remove the effect of leaf structures, and the red wavelength in NDVI is not included in robust wavelength regions in this study.

## Conclusions

Reflectance from adaxial and abaxial leaf surfaces sampled from five woody tree species and two liana species obtained during three growing seasons was measured in order to determine the correlation between LCC and different spectral indices. The reflectance from the adaxial surface was lower than that from the abaxial surface in the visible wavelength, but the opposite was observed in the near-infrared wavelength for all the plant species measured. Very few changes in reflectance were associated with the red-edge spectral region. The MDATT-formatted index was the most effective for estimating LCC, regardless of whether the reflectance data was obtained from the adaxial or abaxial leaf surfaces or a combination thereof. MDATT is an optimal index because it can remove the effects of the leaf outer surface and internal structures when assessing the LCC of several different plant species from different seasons. The red-edge to near-infrared wavelength (723–885 nm) for λ_1_, as well as the red-edge region (697–771 nm) for λ_2_ or λ_3_, constituted robust wavelength regions for constructing the MDATT index for estimating LCC because each combination for MDATT in this region was correlated with LCC with an R^2^ higher than 0.8, regardless of the measured leaf surface or plant species. However, the R^2^ could not reach 0.8 when λ_1_ equaled λ_3_ or λ_2_ equaled λ_3_.

## Additional files

**Additional file 1: Table S1.** Relationships between vegetation indices and LCC for individual plant species (only the top 15 vegetation indices that had a high R^2^ value were listed).

**Additional file 2: Figure S1.** Contour maps for R^2^ between LCC and the MDATT index with the combination of λ_1_ and λ_3_ derived from the adaxial leaf reflectance for each plant species (the dots represent the wavebands combination with highest R^2^. Two combinations have the same highest R^2^ value because λ_2_ and λ_3_ are exchangeable in the MDATT index. a, Chinese elm; b, Virginia creeper; c, torch tree; d, Manchurian lilac; e, grapevine; f, Narrow-leaved oleaster; g, white poplar).

**Additional file 3: Figure S2.** Contour maps for R^2^ between LCC and the MDATT index with the combination of λ_2_ and λ_3_ derived from the adaxial leaf reflectance for each plant species (the dots and letters represent the same as items in Figure S1).

**Additional file 4: Figure S3.** Contour maps for R^2^ between LCC and the MDATT index with the combination of λ_2_ and λ_3_ derived from the abaxial leaf reflectance for each plant species (the dots and letters represent the same items as in Figure S1).

**Additional file 5: Figure S4.** Contour maps for R^2^ between LCC and the MDATT index with the combination of λ_2_ and λ_3_ derived from the abaxial leaf reflectance for each plant species (the dots and letters represent the same items as in Figure S1).

**Additional file 6: Figure S5.** Contour maps for R^2^ between LCC and the MDATT index with the combination of λ_2_ and λ_3_ derived from the adaxial and abaxial leaf reflectance for each plant species (the dots and letters represent the same items as in Figure S1).

**Additional file 7: Figure S6.** Contour maps for R^2^ between LCC and the MDATT index with the combination of λ_2_ and λ_3_ derived from the adaxial and abaxial leaf reflectance for each plant species (the dots and letters represent the same items as in Figure S1).

## Abbreviations

LCC: leaf chlorophyll content
RMSE: root mean square error
MDATT: Modified Datt
SR: single ratio
SD: single difference
ND: normalized difference
NDVI: normalized difference vegetation index
NIR: near infrared
LAI: leaf area index

## References

[1] Gitelson AA, Gritz Y, Merzlyak MN (2003). Relationships between leaf chlorophyll content and spectral reflectance and algorithms for non-destructive chlorophyll assessment in higher plant leaves. J Plant Physiol.

[2] Zhang J, Huang W, Zhou Q (2014). Reflectance variation within the in-chlorophyll centre waveband for robust retrieval of leaf chlorophyll content. PLoS ONE.

[3] Hendry GAF, Brown SB (1987). Tansley Review No. 11. The degradation of chlorophyll-A biological enigma. New Phytol.

[4] Merzlyak MN, Gitelson A (1995). Why and what for the leaves are yellow in autumn? On the interpretation of optical spectra of senescing leaves (*Acer platanoides* L.). J Plant Physiol.

[5] Peñuelas J, Filella I (1998). Visible and near-infrared reflectance techniques for diagnosing plant physiological status. Trends Plant Sci.

[6] Merzlyak MN, Gitelson AA, Chivkunova OB, Rakitin VY (1999). Non-destructive optical detection of pigment changes during leaf senescence and fruit ripening. Physiol Plant.

[7] Manlenovsky Z, Mishra KB, Zeme F, Rascher U, Nedbal L (2009). Scientific and technical challenges in remote sensing of plant canopy reflectance and fluorescence. J Exp Bot.

[8] Tucker CJ (1979). Red and photographic infrared linear combinations for monitoring vegetation. Remote Sens Environ.

[9] Main R, Cho MA, Mathieu R, O’Kennedy MM, Ramoelo A, Koch S (2001). An investigation into robust spectral indices for leaf chlorophyll estimation. ISPRS J Photogramm Remote Sens.

[10] Baret F, Guyot G (1991). Potential and limits of vegetation indices for LAI and APAR assessment. Remote Sens Environ.

[11] Huete AR, Hua G, Qi J, Chehbouni A, Van Leeuwen WJD (1992). Normalization of multidirectional red and NIR reflectances with the SAVI. Remote Sens Environ.

[12] Qi J, Moran MS, Cabot F, Dedieu G (1995). Normalization of sun/view angle effects using spectral albedo-based vegetation indices. Remote Sens Environ.

[13] Mutanga O, Skidmore AK (2004). Hyperspectral band depth analysis for a better estimation of grass biomass (*Cenchrus ciliaris*) measured under controlled laboratory conditions. Int J Appl Earth Obs.

[14] Yoder BJ, Pettigrew-Crosby RE (1995). Predicting nitrogen and chlorophyll content and concentrations from reflectance spectra (400–2500 nm) at leaf and canopy scales. Remote Sens Environ.

[15] Carter GA (1994). Ratios of leaf reflectances in narrow wavebands as indicators of plant stress. Int J Remote Sens.

[16] Peñuelas J, Baret F, Filella I (1995). Semiempirical indexes to assess carotenoids chlorophyll-a ratio from leaf spectral reflectance. Photosynthetica.

[17] Blackburn GA (1998). Quantifying chlorophylls and carotenoids at leaf and canopy scales: an evaluation of some hyperspectral approaches. Remote Sens Environ.

[18] Blackburn GA (1998). Spectral indices for estimating photosynthetic pigment concentrations: a test using senescent tree leaves. Int J Remote Sens.

[19] Sims DA, Gamon JA (2002). Relationships between leaf pigment content and spectral reflectance across a wide range of species, leaf structures and developmental stages. Remote Sens Environ.

[20] Gitelson AA, Viña A, Ciganda V, Rundquist DC, Arkebauer TJ (2005). Remote estimation of canopy chlorophyll content in crops. Geophys Res Lett.

[21] Datt B (1999). A new reflectance index for remote sensing of chlorophyll content in higher plants: tests using Eucalyptus leaves. J Plant Physiol.

[22] Datt B (1999). Visible/near infrared reflectance and chlorophyll content in Eucalyptus leaves. Int J Remote Sens.

[23] Vogelmann JE, Rock BN, Moss DM (1993). Red edge spectral measurements from sugar maple leaves. Int J Remote Sens.

[24] Lucas NS, Curran PJ, Plummer SE, Danson FM (2000). Estimating the stem carbon production of a coniferous forest using ecosystem simulation model driven by the remotely sensed red edge. Int J Remote Sens.

[25] Weiss M, Troufleau D, Baret F, Chauki H, Prevot L, Olioso A, Bruguier N, Brisson N (2001). Coupling canopy functioning and radiative transfer models for remote sensing data assimilation. Agric For Meteorol.

[26] Woolley JT (1971). Reflectance and transmittance of light by leaves. Plant Physiol.

[27] Baldini E, Facini O, Nerozzi F, Rossi F, Rotondi A (1997). Leaf characteristics and optical properties of different woody species. Trees Struct Funct.

[28] Lu S, Lu X, Zhao W, Liu Y, Wang Z, Omasa K (2015). Comparing vegetation indices for remote chlorophyll measurement of white poplar and Chinese elm leaves with different adaxial and abaxial surfaces. J Exp Bot.

[29] Maire GL, Francois C, Dufrene E (2004). Towards universal broad leaf chlorophyll indices using PROSPECT simulated database and hyperspectral reflectance measurements. Remote Sens Environ.

[30] Wintermans JF, De Mots A (1965). Spectrophotometric characteristics of chlorophylls a and b and their phenophytins in ethanol. Biochem Biophys Acta.

[31] Gitelson AA, Merzlyak MN (1996). Signature analysis of leaf reflectance spectra: algorithm development for remote sensing of chlorophyll. J Plant Physiol.

[32] Lichtenthaler HK (1996). Non-destructive determination of chlorophyll content of leaves of a green and an aurea mutant of tobacco by reflectance measurements. J Plant Physiol.

[33] Datt B (1998). Remote sensing of chlorophyll a, chlorophyll b, chlorophyll a + b, and total carotenoid content in eucalyptus leaves. Remote Sens Environ.

[34] Zarco-Tejada PJ, Miller JR, Noland TL, Mohammed GH, Sampson PH (2001). Scaling-up and model inversion methods with narrowband optical indices for chlorophyll content estimation in closed forest canopies with hyperspectral data. IEEE Trans Geosci Remote Sens.

[35] Zarco-Tejada PJ, Berjón A, López-Lozano R, Miller JR, Martín P, Cachorro V, González MR, De Frutos A (2005). Assessing vineyard condition with hyperspectral indices: leaf and canopy reflectance simulation in a row-structured discontinuous canopy. Remote Sens Environ.

[36] Zhu Y, Zhou D, Yao Y, Cao W (2007). Quantitative relationships of leaf nitrogen status to canopy spectral reflectance in rice. Aust J Agric Res.

[37] Gitelson AA, Merzlyak MN (1994). Spectral reflectance changes associated with autumn senescence of *Aesculus hippocastanum* L. and *Acer platanoides* L. leaves. Spectral features and relation to chlorophyll estimation. J Plant Physiol.

[38] Takebe M, Yoneyama T (1989). Measurement of leaf color scores and its implication to nitrogen nutrition of rice plants. Jpn Agric Res.

[39] Richardson AD, Duigan SP, Berlyn GP (2002). An evaluation of noninvasive methods to estimate foliar chlorophyll content. New Phytol.

[40] Mutanga O, Skidmore AK (2007). Red edge shift and biochemical content in grass canopies. ISPRS J Photogramm Remote Sens.

[41] Kim MS, Daughtry C, Chappelle E, McMurtrey J, Walthall C. The use of high spectral resolution bands for estimating absorbed photosynthetically active radiation (APAR). In; Proceedings 6th international symposium on physical measurements and signatures in remote sensing. Val d’Isere France; 1994. p. 299–306.

[42] Daughtry CST, Walthall CL, Kim MS, De Colstoun EB, McMurtrey JE (2000). Estimating corn leaf chlorophyll concentration from leaf and canopy reflectance. Remote Sens Environ.

[43] Rondeaux G, Steven M, Baret F (1996). Optimization of soil-adjusted vegetation indices. Remote Sens Environ.

[44] Haboudane D, Miller JR, Tremblay N, Zarco-Tejada PJ, Dextraze L (2002). Integrated narrow-band vegetation indices for prediction of crop chlorophyll content for application to precision agriculture. Remote Sens Environ.

[45] Tian YC, Yao X, Yang J, Cao WX, Hannaway DB, Zhu Y (2011). Assessing newly developed and published vegetation indices for estimating rice leaf nitrogen concentration with ground- and space-based hyperspectral reflectance. Field Crops Res.

[46] Gates DM, Keegan HJ, Schleter JC, Weidner VR (1965). Spectral properties of plants. Appl Opt.

[47] Vogelmann TC, Martin G (1993). The functional significance of palisade tissue: penetration of directional versus diffuse light. Plant, Cell Environ.

[48] Stuckens J, Verstraeten WW, Delalieux S, Swennen R, Coppin P (2009). A dorsiventral leaf radiative transfer model: development, validation and improved model inversion techniques. Remote Sens Environ.

[49] Shigematsu S. The effect of pH on color behavior of anthocyanin. Research Note of Ehime Education Center; 2007. p. 39–42 **(in Japanese)**.

[50] Baret F, Andrieu B, Guyot G, Lichtenthaler HK (1988). A simple model for leaf optical properties in visible and near-infrared: application to the analysis of spectral shifts determinism. Applications of chlorophyll fluorescence in photosynthesis research, stress physiology, hydrobiology and remote sensing.

[51] Curran PJ, Dungan JL, Gholz HL (1990). Exploring the relationship between reflectance red edge and chlorophyll content in slash pine. Tree Physiol.

