# Supplementary material for: A robust vegetation index for remotely assessing chlorophyll content of dorsiventral leaves across several species in different seasons
Source: Plant Methods. 2018 Feb 14;14:15. doi: 10.1186/s13007-018-0281-z (PMC5812224; doi:10.1186/s13007-018-0281-z)
Supplement: Supplementary file 1 — Additional file 1: Table S1. Relationships between vegetation indices and LCC for individual plant species (only the top 15 vegetation indices that had a high R2 value were listed). [file 13007_2018_281_MOESM1_ESM.docx]

Table S1. Relationships between vegetation indices and LCC for individual plant species (only the top 15 vegetation indices that had a high R^2^ value were listed).

| **White poplar** | | | | | | | | |
| --- | --- | --- | --- | --- | --- | --- | --- | --- |
| Adaxial and abaxial | | | adaxial | | | abaxial | | |
| VI | R^2^ | RMSE  (μg/cm^2^) | VI | R^2^ | RMSE  (μg/cm^2^) | VI | R^2^ | RMSE  (μg/cm^2^) |
| **MDATT: (R_722_-R_745_)/(R_722_-R_723_)** | 0.859 | 5.875 | **MDATT: (R_618_-R_711_)/(R_618_-R_641_)** | 0.955 | 3.365 | **MDATT: (R_622_-R_971_)/(R_622_-R_639_)** | 0.937 | 3.968 |
| (R_719_-R_726_)/(R_719_-R_743_) | 0.805 | 6.904 | SR: R_750_/R_557_ | 0.953 | 3.491 | D_754_/D_704_ | 0.930 | 4.182 |
| SD: R_520_-R_522_ | 0.747 | 7.873 | R_750_/R_550_ | 0.952 | 3.474 | (R_719_-R_726_)/(R_719_-R_743_) | 0.835 | 6.414 |
| ND: (R_608_-R_689_)/(R_608_+R_689_) | 0.705 | 8.497 | R_860_/R_550_ | 0.949 | 3.561 | SD: R_734_-R_960_ | 0.802 | 7.036 |
| D_754_/D_704_ | 0.690 | 8.714 | VOG2 | 0.942 | 3.812 | SR: R_981_/R_735_ | 0.742 | 8.020 |
| TCARI | 0.669 | 9.001 | 1/R_550_-1/R_750_ | 0.940 | 3.857 | ND: (R_735_-R_981_)/(R_735_+R_981_) | 0.739 | 8.069 |
| R_672_/(R_550_*R_708_) | 0.648 | 9.286 | D_754_/D_704_ | 0.934 | 4.046 | R_672_/(R_550_*R_708_) | 0.739 | 8.070 |
| SR: R_607_/R_687_ | 0.645 | 9.320 | RII | 0.932 | 4.131 | TCARI: | 0.726 | 8.270 |
| MCARI | 0.528 | 10.745 | R_750_/R_710_ | 0.930 | 4.168 | (R_850_-R_710_)/(R_850_-R_680_) | 0.685 | 8.861 |
| (R_850_-R_710_)/(R_850_-R_680_) | 0.514 | 10.914 | ND: (R_730_-R_969_)/(R_730_+R_969_) | 0.924 | 4.365 | TCARI | 0.615 | 9.798 |
| R_450_/R_550_ | 0.399 | 12.129 | R_860_/(R_550_*R_708_) | 0.922 | 4.417 | D_740_ | 0.526 | 10.871 |
| VOG2 | 0.324 | 12.863 | R_750_/R_700_ | 0.909 | 4.755 | TCARI/OSAVI | 0.522 | 10.922 |
| D_740_ | 0.317 | 12.931 | SD: R_732_-R_969_ | 0.908 | 4.782 | R_450_/R_550_ | 0.515 | 11.000 |
| TCARI/OSAVI | 0.269 | 13.382 | 1/R_700_ | 0.907 | 4. 807 | R_672_/R_550_ | 0.514 | 11.014 |
| R_672_/R_550_ | 0.269 | 13.383 | R_672_/(R_550_*R_708_) | 0.906 | 4.839 | VOG2 | 0.433 | 11.891 |

Table S1 (continued)

| **Narrow-leaved oleaster** | | | | | | | | |
| --- | --- | --- | --- | --- | --- | --- | --- | --- |
| Adaxial and abaxial | | | adaxial | | | abaxial | | |
| VI | R^2^ | RMSE  (μg/cm^2^) | VI | R^2^ | RMSE  (μg/cm^2^) | VI | R^2^ | RMSE  (μg/cm^2^) |
| **MDATT: (R_723_-R_735_)/(R_723_-R_726_)** | 0.936 | 5.588 | SR: R_742_/R_741_ | 0.981 | 3.090 | **MDATT: (R_695_-R_745_)/(R_695_-R_740_)** | 0.962 | 4.323 |
| (R_719_-R_726_)/(R_719_-R_743_) | 0.931 | 5.813 | ND: (R_741_-R_742_)/(R_741_+R_742_) | 0.981 | 3.097 | (R_719_-R_726_)/(R_719_-R_743_) | 0.937 | 5.583 |
| (R_850_-R_710_)/(R_850_-R_680_) | 0.784 | 10.278 | **MDATT: (R_698_-R_742_)/(R_698_-R_741_)** | 0.980 | 3.131 | D_754_/D_704_ | 0.930 | 5.899 |
| SD: R_463_-R_540_ | 0.769 | 10.616 | VOG2 | 0.978 | 3.327 | SD: R_752_-R_754_ | 0.914 | 6.530 |
| TCARI | 0.769 | 10.622 | R_750_/R_710_ | 0.977 | 3.395 | (R_850_-R_710_)/(R_850_-R_680_) | 0.885 | 7.548 |
| D_754_/D_704_ | 0.696 | 12.191 | SD: R_728_-R_967_ | 0.974 | 3.566 | D_740_ | 0.875 | 7.900 |
| ND: (R_550_-R_553_)/(R_550_+R_553_) | 0.645 | 13.162 | (R_719_-R_726_)/(R_719_-R_743_) | 0.968 | 3.988 | SR: R_752_/R_754_ | 0.865 | 8.205 |
| R_672_/(R_550_*R_708_) | 0.548 | 14.853 | R_750_/R_700_ | 0.967 | 4.046 | ND: (R_752_-R_754)_/(R_752_+R_754_) | 0.865 | 8.206 |
| TCARI/OSAVI | 0.548 | 14.864 | RII | 0.967 | 4.051 | TCARI | 0.831 | 9.178 |
| SR: R_616_/R_439_ | 0.523 | 15.270 | D_740_ | 0.965 | 4.160 | MCARI | 0.823 | 9.396 |
| MCARI | 0.505 | 15.550 | R_860_/R_550_ | 0.960 | 4.444 | D_730_ | 0.811 | 9.704 |
| D_740_ | 0.459 | 16.252 | R_750_/R_550_ | 0.960 | 4.444 | VOG2 | 0.805 | 9.839 |
| D_730_ | 0.419 | 16.848 | 1/R_700_-1/R_750_ | 0.952 | 4.894 | R_450_/R_550_ | 0.767 | 10.764 |
| VOG2 | 0.406 | 17.038 | 1/R_700_ | 0.944 | 5.269 | TCARI/OSAVI | 0.712 | 11.964 |
| R_750_/R_710_ | 0.316 | 18.273 | D_730_ | 0.943 | 5.331 | R_672_/R_550_ | 0.615 | 13.842 |

Table S1 (continued)

| **Manchurian lilac** | | | | | | | | |
| --- | --- | --- | --- | --- | --- | --- | --- | --- |
| Adaxial and abaxial | | | adaxial | | | abaxial | | |
| VI | R^2^ | RMSE  (μg/cm^2^) | VI | R^2^ | RMSE  (μg/cm^2^) | VI | R^2^ | RMSE  (μg/cm^2^) |
| **MDATT: (R_726_-R_738_)/(R_726_-R_729_)** | 0.888 | 5.573 | SR: R_930_/ R_557_ | 0.945 | 3.936 | **MDATT: (R_726_-R_738_)/(R_726_-R_730_)** | 0.916 | 4.864 |
| (R_719_-R_726_)/(R_719_-R_743_) | 0.866 | 6.081 | R_860_/R_550_ | 0.944 | 3.979 | VOG2 | 0.904 | 5.204 |
| ND: (R_742_-R_743_)/(R_742_+R_743_) | 0.719 | 8.820 | R_750_/R_550_ | 0.939 | 4.142 | SR: R_742_/ R_741_ | 0.901 | 5.269 |
| SR: R_743_/ R _741_ | 0.718 | 8.834 | R_860_/(R_550_*R_708_) | 0.938 | 4.163 | ND: (R_741_-R_742_)/(R_741_+R_742_) | 0.901 | 5.274 |
| (R_850_-R_710_)/(R_850_-R_680_) | 0.676 | 9.479 | **MDATT: (R_422_-R_929_)/(R_422_-R_704_)** | 0.938 | 4.164 | SD:R_741_-R_742_ | 0.889 | 5.593 |
| SD: R_741_-R_743_ | 0.673 | 9.524 | R_750_/R_710_ | 0.935 | 4.273 | (R_719_-R_726_)/(R_719_-R_743_) | 0.883 | 5.715 |
| D_754_/D_704_ | 0.666 | 9.624 | VOG2 | 0.933 | 4.331 | RII | 0.882 | 5.768 |
| D_740_ | 0.662 | 9.679 | 1/R_550_-1/R_750_ | 0.924 | 4.627 | D_740_ | 0.878 | 5.862 |
| VOG2 | 0.653 | 9.798 | ND: (R_741_-R_742_)/(R_741_+R_742_) | 0.924 | 4.631 | R_750_/R_710_ | 0.865 | 6.167 |
| D_730_ | 0.618 | 10.283 | RII | 0.924 | 4.639 | R_800_/R_650_ | 0.854 | 6.406 |
| NDI | 0.558 | 11.062 | 1/R_700_-1/R_750_ | 0.922 | 4.687 | D_754_/D_704_ | 0.851 | 6.470 |
| R_705_/(R_717_+R_491_) | 0.551 | 11.153 | R_750_/R_700_ | 0.922 | 4.699 | PSSRb | 0.844 | 6.628 |
| R_750_/R_710_ | 0.541 | 11.277 | 1/R_700_ | 0.918 | 4.815 | D_730_ | 0.839 | 6.732 |
| R_450_/R_550_ | 0.532 | 11.389 | (R_719_-R_726_)/(R_719_-R_743_) | 0.894 | 5.469 | R_800_/R_650_ | 0.826 | 7.002 |
| TCARI: | 0.520 | 11.527 | SD: R_741_-R_742_ | 0.893 | 5.501 | R_750_/R_700_ | 0.826 | 7.004 |

Table S1 (continued)

| **Chinese elm** | | | | | | | | |
| --- | --- | --- | --- | --- | --- | --- | --- | --- |
| Adaxial and abaxial | | | adaxial | | | abaxial | | |
| VI | R^2^ | RMSE  (μg/cm^2^) | VI | R^2^ | RMSE  (μg/cm^2^) | VI | R^2^ | RMSE  (μg/cm^2^) |
| **MDATT: (R_719_-R_729_)/(R_719_-R_720_)** | 0.927 | 4.549 | **MDATT: (R_682_-R_728_)/(R_682_-R_726_)** | 0.959 | 3.443 | **MDATT: (R_686_-R_738_)/(R_686_-R_739_)** | 0.963 | 3.277 |
| (R_719_-R_726_)/(R_719_-R_743_) | 0.834 | 6.842 | SR: R_733_/R_735_ | 0.954 | 3.641 | SR: R_743_/R_741_ | 0.957 | 3.515 |
| SD: R_498_-R_709_ | 0.824 | 7.056 | ND: (R_733_-R_735_)/(R_733_+R_735_) | 0.954 | 3.644 | ND: (R_741_-R_743_)/(R_741_+R_743_) | 0.957 | 3.518 |
| ND: (R_407_-R_704_)/(R_407_+R_704_) | 0.814 | 7.251 | SD: R_505_-R_710_ | 0.946 | 3.948 | VOG2:(R_734_-R_747_)/(R_715_+R_726_) | 0.955 | 3.602 |
| (R_850_-R_710_)/(R_850_-R_680_) | 0.756 | 8.301 | R_750_/R_710_ | 0.944 | 3.991 | SD: R_741_-R_743_ | 0.944 | 3.998 |
| SR: R_705_/R_407_ | 0.746 | 8.469 | D_740_ | 0.938 | 4.217 | D_740_ | 0.940 | 4.152 |
| R_450_/R_550_ | 0.683 | 9.468 | VOG2 | 0.935 | 4.325 | R_750_/R_710_ | 0.919 | 4.829 |
| D_740_ | 0.682 | 9.470 | 1/R_700_-1/R_750_ | 0.929 | 4.520 | RII | 0.909 | 5.111 |
| TCARI | 0.651 | 9.922 | 1/R_700_ | 0.927 | 4.568 | (R_719_-R_726_)/(R_719_-R_743_) | 0.905 | 5.220 |
| D_754_/D_704_ | 0.648 | 9.969 | R_750_/R_700_ | 0.923 | 4.685 | D_730_ | 0.903 | 5.274 |
| VOG2 | 0.646 | 10.001 | RII | 0.920 | 4.796 | R_860_/R_550_ | 0.898 | 5.406 |
| D_730_ | 0.642 | 10.056 | R_750_/R_550_ | 0.919 | 4.806 | (R_850_-R_710_)/(R_850_-R_680_) | 0.897 | 5.422 |
| TCARI/OSAVI | 0.618 | 10.394 | (R_850_-R_710_)/(R_850_-R_680_) | 0.914 | 4.959 | R_860_/(R_550_*R_708_) | 0.889 | 5.654 |
| MCARI | 0.610 | 10.493 | D_730_ | 0.912 | 5.030 | R_750_/R_550_ | 0.887 | 5.695 |
| R_672_/(R_550_*R_708_) | 0.552 | 11.252 | R_860_/R_550_ | 0.910 | 5.089 | D_754_/D_704_ | 0.869 | 6.136 |

Table S1 (continued)

| **Virgineer creeper** | | | | | | | | |
| --- | --- | --- | --- | --- | --- | --- | --- | --- |
| Adaxial and abaxial | | | adaxial | | | abaxial | | |
| VI | R^2^ | RMSE  (μg/cm^2^) | VI | R^2^ | RMSE  (μg/cm^2^) | VI | R^2^ | RMSE  (μg/cm^2^) |
| **MDATT: (R_718_-R_723_)/(R_718_-R_731_)** | 0.945 | 4.105 | **MDATT: (R_482_-R_527_)/(R_482_-R_519_)** | 0.973 | 2.922 | **MDATT: (R_650_-R_716_)/(R_650_-R_653_)** | 0.970 | 3.033 |
| (R_719_-R_726_)/(R_719_-R_743_) | 0.914 | 5.115 | R_737_-R_739_ | 0.971 | 3.018 | R_737_-R_738_ | 0.960 | 3.513 |
| (R_850_-R_710_)/(R_850_-R_680_) | 0.840 | 6.985 | D_740_ | 0.970 | 3.069 | D_740_ | 0.959 | 3.575 |
| ND: (R_737_-R_738_)/(R_737_+R_738_) | 0.828 | 7.257 | R_445_/ R_616_ | 0.969 | 3.099 | R_733_/R_732_ | 0.954 | 3.764 |
| SD: R_434_-R_705_ | 0.799 | 7.830 | (R_451_-R_606_)/(R_451_+R_606_) | 0.963 | 3.371 | (R_732_-R_733_)/(R_732_+R_733_) | 0.954 | 3.764 |
| SR: R_751_/ R_730_ | 0.799 | 7.830 | D_730_ | 0.948 | 4.009 | R_750_/R_710_ | 0.939 | 4.339 |
| D_740_ | 0.780 | 8.199 | (R_719_-R_726_)/(R_719_-R_743_) | 0.947 | 4.045 | VOG2:(R_734_-R_747_)/(R_715_+R_726_) | 0.935 | 4.481 |
| VOG2 | 0.745 | 8.827 | R_750_/R_700_ | 0.938 | 4.380 | D_730_ | 0.932 | 4.611 |
| D_730_ | 0.737 | 8.964 | (R_850_-R_710_)/(R_850_-R_680_) | 0.935 | 4.480 | RII | 0.929 | 4.703 |
| NDI | 0.735 | 8.988 | NDI | 0.933 | 4.552 | (R_850_-R_710_)/(R_850_-R_680_) | 0.911 | 5.268 |
| R_705_/(R_717_+R_491_) | 0.715 | 9.334 | R_750_/R_710_ | 0.933 | 4.574 | R_800_/R_650_ | 0.908 | 5.338 |
| R_750_/R_710_ | 0.681 | 9.868 | R_800_/R_650_ | 0.921 | 4.959 | (R_719_-R_726_)/(R_719_-R_743_) | 0.907 | 5.383 |
| 1/R_700_ | 0.650 | 10.339 | 1/R_700_-1/R_750_ | 0.920 | 4.999 | R_750_/R_700_ | 0.907 | 5.387 |
| D_754_/D_704_ | 0.644 | 10.430 | VOG2:(R_734_-R_747_)/(R_715_+R_726_) | 0.912 | 5.216 | 1/R_700_-1/R_750_ | 0.892 | 5.800 |
| 1/R_700_-1/R_750_ | 0.632 | 10.605 | 1/R_700_ | 0.912 | 5.239 | NDI | 0.888 | 5.891 |

Table S1 (continued)

| **Grapevine** | | | | | | | | |
| --- | --- | --- | --- | --- | --- | --- | --- | --- |
| Adaxial and abaxial | | | adaxial | | | abaxial | | |
| VI | R^2^ | RMSE  (μg/cm^2^) | VI | R^2^ | RMSE  (μg/cm^2^) | VI | R^2^ | RMSE  (μg/cm^2^) |
| **MDATT: (R_521_-R_744_)/(R_521_-R_743_)** | 0.833 | 2.771 | SR: R_406_/R_609_ | 0.892 | 2.244 | **MDATT: (R_686_-R_949_)/(R_686_-R_708_)** | 0.882 | 2.346 |
| (R_719_-R_726_)/(R_719_-R_743_) | 0.801 | 3.025 | **MDATT: (R_409_-R_712_)/(R_409_-R_699_)** | 0.885 | 2.317 | SR: R_744_/R_743_ | 0.864 | 2.522 |
| ND: (R_743_-R_744_)/(R_743_+R_744_) | 0.748 | 3.401 | 1/R_700_-1/R_750_ | 0.876 | 2.406 | ND: (R_743_-R_744_)/(R_743_+R_744_) | 0.864 | 2.522 |
| SR: R_744_/R_743_ | 0.748 | 3.402 | ND: (R_746_-R_747_)/(R_746_+R_747_) | 0.858 | 2.576 | VOG2 | 0.861 | 2.545 |
| (R_850_-R_710_)/(R_850_-R_680_) | 0.739 | 3.461 | R_750_/R_710_ | 0.847 | 2.676 | R_750_/R_710_ | 0.816 | 2.933 |
| VOG2 | 0.705 | 3.680 | R_750_/R_700_ | 0.843 | 2.711 | (R_719_-R_726_)/(R_719_-R_743_) | 0.804 | 3.025 |
| D_754_/D_704_ | 0.701 | 3.702 | VOG2 | 0.841 | 2.722 | (R_850_-R_710_)/(R_850_-R_680_) | 0.801 | 3.048 |
| SD: R_746_-R_747_ | 0.643 | 4.045 | R_750_/R_550_ | 0.832 | 2.801 | SD: R_743_-R_745_ | 0.770 | 3.274 |
| R_750_/R_710_ | 0.637 | 4.084 | (R_850_-R_710_)/(R_850_-R_680_) | 0.826 | 2.849 | R_860_/R_550_ | 0.762 | 3.334 |
| NDI | 0.626 | 4.145 | NDI | 0.821 | 2.894 | D_740_ | 0.759 | 3.355 |
| D_740_ | 0.620 | 4.175 | (R_719_-R_726_)/(R_719_-R_743_) | 0.818 | 2.914 | RII | 0.750 | 3.416 |
| R_705_/(R_717_+R_491_) | 0.613 | 4.216 | R_800_/R_650_ | 0.814 | 2.947 | NDI | 0.743 | 3.462 |
| D_730_ | 0.586 | 4.360 | R_860_/R_550_ | 0.804 | 3.022 | R_800_/R_650_ | 0.742 | 3.471 |
| TCARI/OSAVI | 0.561 | 4.490 | 1/R_700_ | 0.801 | 3.046 | R_750_/R_700_ | 0.740 | 3.481 |
| 1/R_700_-1/R_750_ | 0.549 | 4.549 | R_860_/(R_550_*R_708_) | 0.795 | 3.090 | D_754_/D_704_ | 0.738 | 3.500 |

Table S1 (continued)

| **Torch tree** | | | | | | | | |
| --- | --- | --- | --- | --- | --- | --- | --- | --- |
| Adaxial and abaxial | | | adaxial | | | abaxial | | |
| VI | R^2^ | RMSE  (μg/cm^2^) | VI | R^2^ | RMSE  (μg/cm^2^) | VI | R^2^ | RMSE  (μg/cm^2^) |
| **MDATT: (R_713_-R_739_)/(R_713_-R_722_)** | 0.878 | 3.491 | **MDATT: (R_694_-R_723_)/(R_694_-R_712_)** | 0.954 | 2.150 | **MDATT: (R_415_-R_749_)/(R_415_-R_737_)** | 0.948 | 2.298 |
| (R_719_-R_726_)/(R_719_-R_743_) | 0.776 | 4.722 | SR: R_408_/R_700_ | 0.953 | 2.179 | VOG2 | 0.904 | 3.112 |
| D_754_/D_704_ | 0.629 | 6.073 | ND: (R_739_-R_740_)/(R_739_+R_740_) | 0.950 | 2.250 | SR: R_746_/R_739_ | 0.900 | 3.190 |
| SD: R_437_-R_706_ | 0.629 | 6.078 | R_750_/R_710_ | 0.943 | 2.409 | SD: R_741_-R_746_ | 0.899 | 3.193 |
| ND: (R_745_-R_746_)/(R_745_+R_746_) | 0.627 | 6.094 | SD: R_745_-R_746_ | 0.943 | 2.412 | ND: (R_742_-R_746_)/(R_742_+R_746_) | 0.898 | 3.212 |
| SR: R_746_/R_745_ | 0.626 | 6.096 | R_750_/R_700_ | 0.942 | 2.428 | D_740_ | 0.892 | 3.307 |
| (R_850_-R_710_)/(R_850_-R_680_) | 0.619 | 6.157 | D_740_ | 0.932 | 2.630 | D_754_/D_704_ | 0.888 | 3.368 |
| VOG2 | 0.554 | 6.664 | VOG2 | 0.928 | 2.696 | D_730_ | 0.812 | 4.362 |
| D_740_ | 0.536 | 6.797 | 1/R_700_-1/R_750_ | 0.927 | 2.724 | (R_719_-R_726_)/(R_719_-R_743_) | 0.780 | 4.724 |
| D_730_ | 0.468 | 7.274 | RII | 0.925 | 2.765 | R_750_/R_710_ | 0.777 | 4.757 |
| R_750_/R_710_ | 0.449 | 7.405 | 1/R_700_ | 0.921 | 2.836 | RII | 0.770 | 4.831 |
| NDI | 0.418 | 7.606 | R_800_/R_650_ | 0.900 | 3.179 | (R_850_-R_710_)/(R_850_-R_680_) | 0.757 | 4.966 |
| R_705_/(R_717_+R_491_) | 0.405 | 7.694 | D_730_ | 0.858 | 3.786 | NDI | 0.679 | 5.702 |
| 1/R_700_ | 0.388 | 7.804 | (R_719_-R_726_)/(R_719_-R_743_) | 0.858 | 3.794 | R_860_/(R_550_*R_708_) | 0.668 | 5.799 |
| 1/R_700_-1/R_750_ | 0.374 | 7.889 | NDI | 0.852 | 3.871 | R_750_/R_700_ | 0.634 | 6.087 |
