# Supplementary figures and images for: A robust vegetation index for remotely assessing chlorophyll content of dorsiventral leaves across several species in different seasons
Source: Plant Methods. 2018 Feb 14;14:15. doi: 10.1186/s13007-018-0281-z (PMC5812224; doi:10.1186/s13007-018-0281-z)

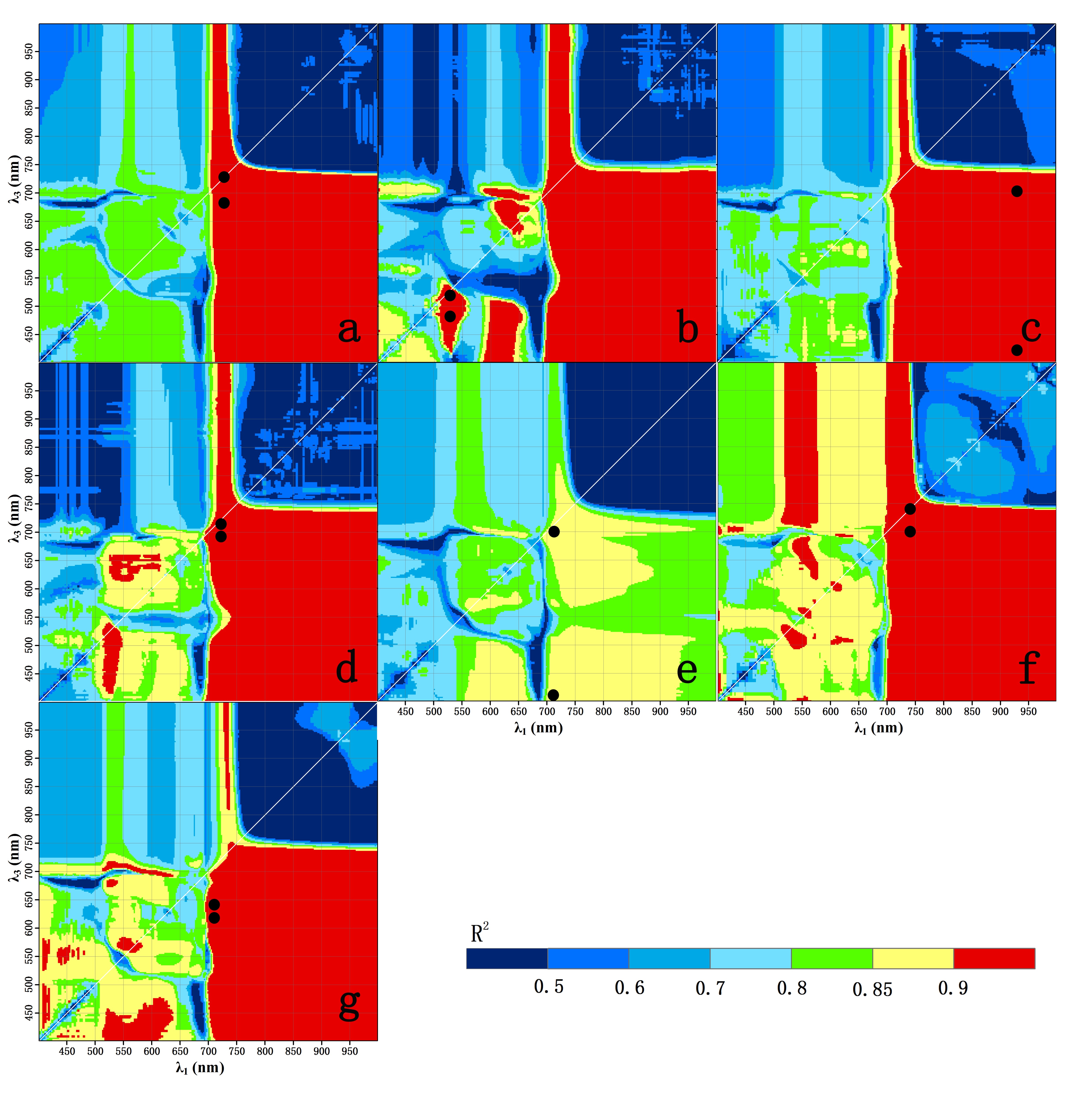

Supplement: Supplementary file 2 — Additional file 2: Figure S1. Contour maps for R2 between LCC and the MDATT index with the combination of λ1 and λ3 derived from the adaxial leaf reflectance for each plant species (the dots represent the wavebands combination with highest R2. Two combinations have the same highest R2 value because λ2 and λ3 are exchangeable in the MDATT index. a, Chinese elm; b, Virginia creeper; c, torch tree; d, Manchurian lilac; e, grapevine; f, Narrow-leaved oleaster; g, white poplar). [file 13007_2018_281_MOESM2_ESM.jpg]

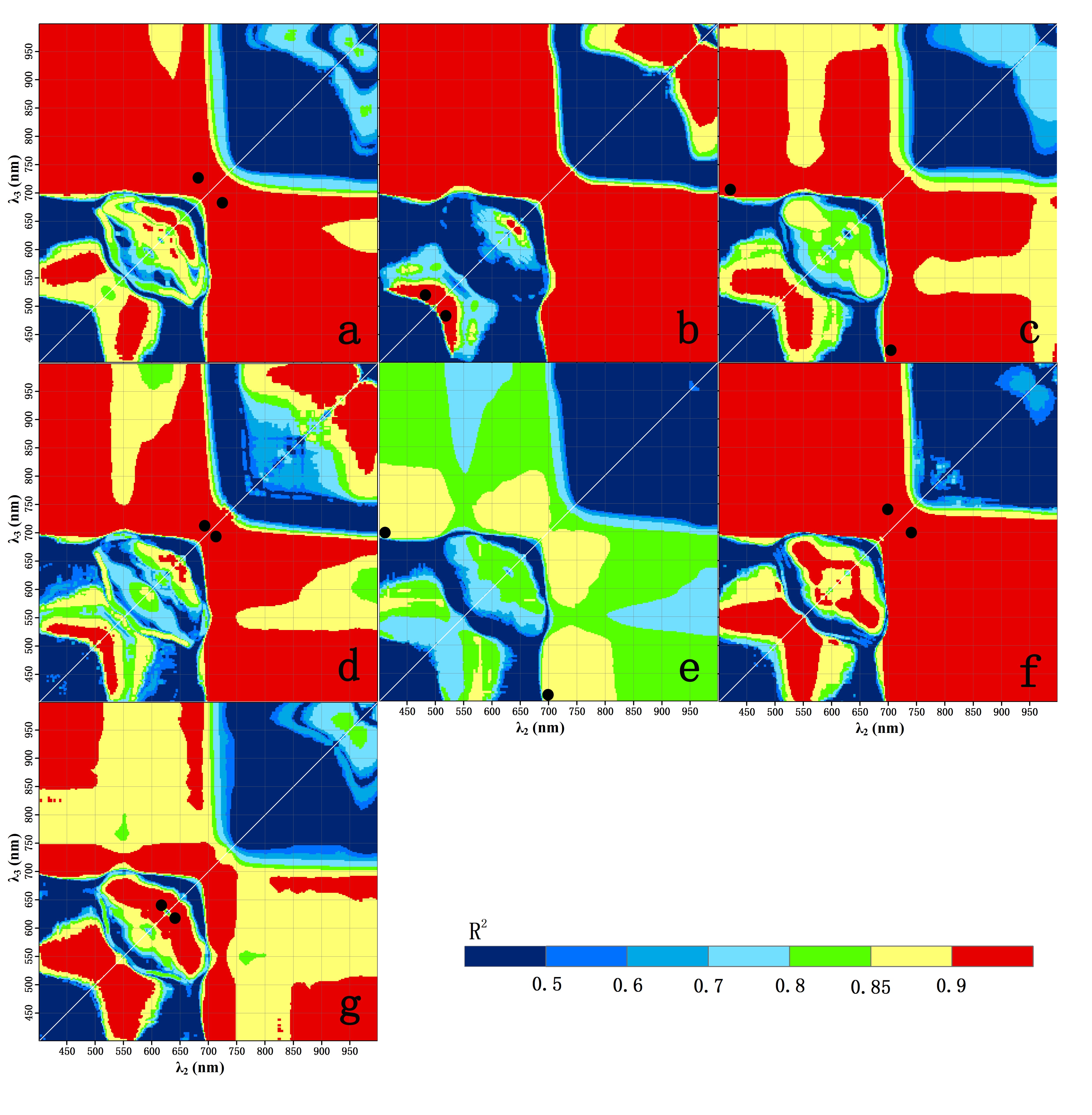

Supplement: Supplementary file 3 — Additional file 3: Figure S2. Contour maps for R2 between LCC and the MDATT index with the combination of λ2 and λ3 derived from the adaxial leaf reflectance for each plant species (the dots and letters represent the same as items in Figure S1). [file 13007_2018_281_MOESM3_ESM.jpg]

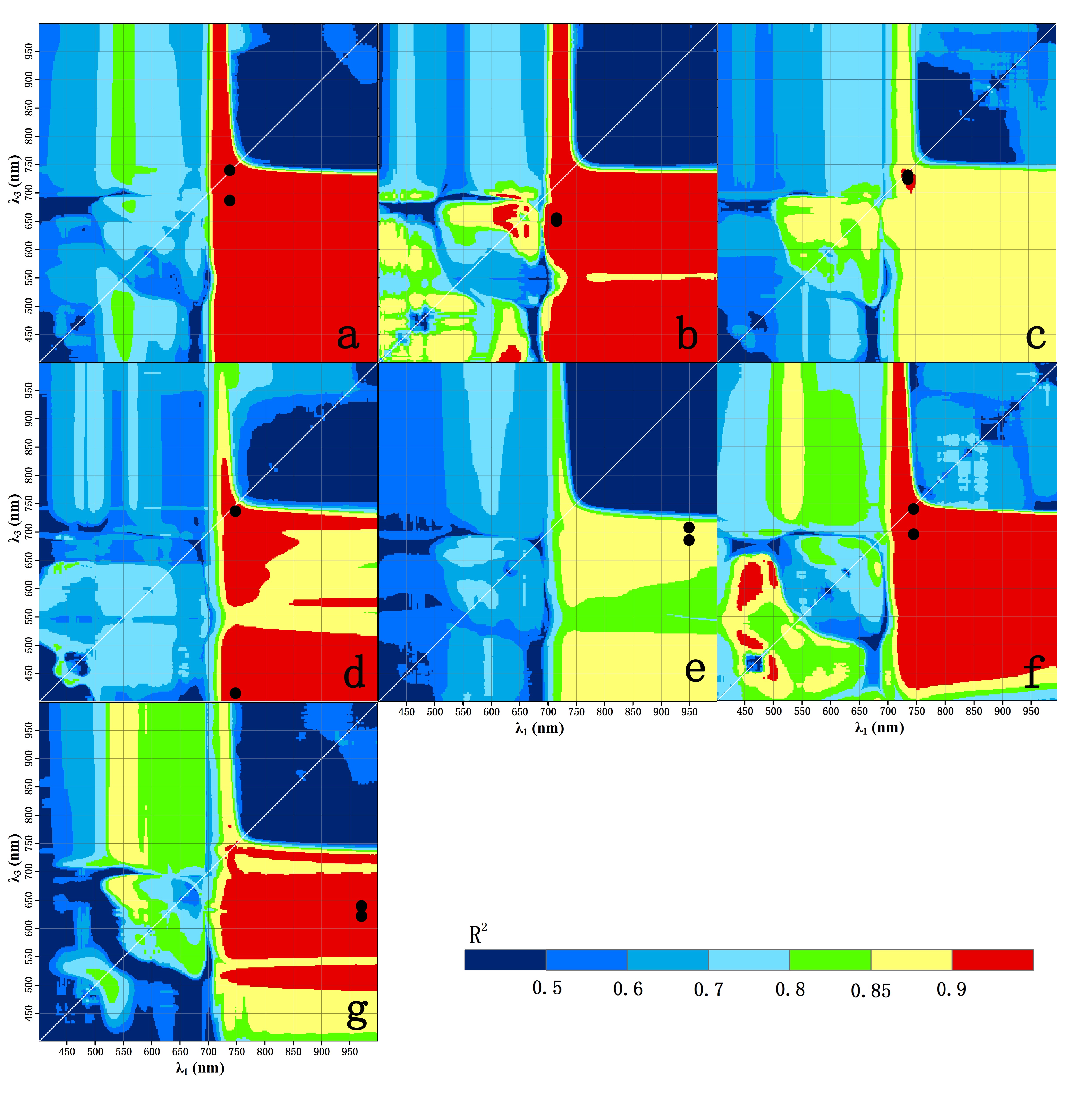

Supplement: Supplementary file 4 — Additional file 4: Figure S3. Contour maps for R2 between LCC and the MDATT index with the combination of λ2 and λ3 derived from the abaxial leaf reflectance for each plant species (the dots and letters represent the same items as in Figure S1). [file 13007_2018_281_MOESM4_ESM.jpg]

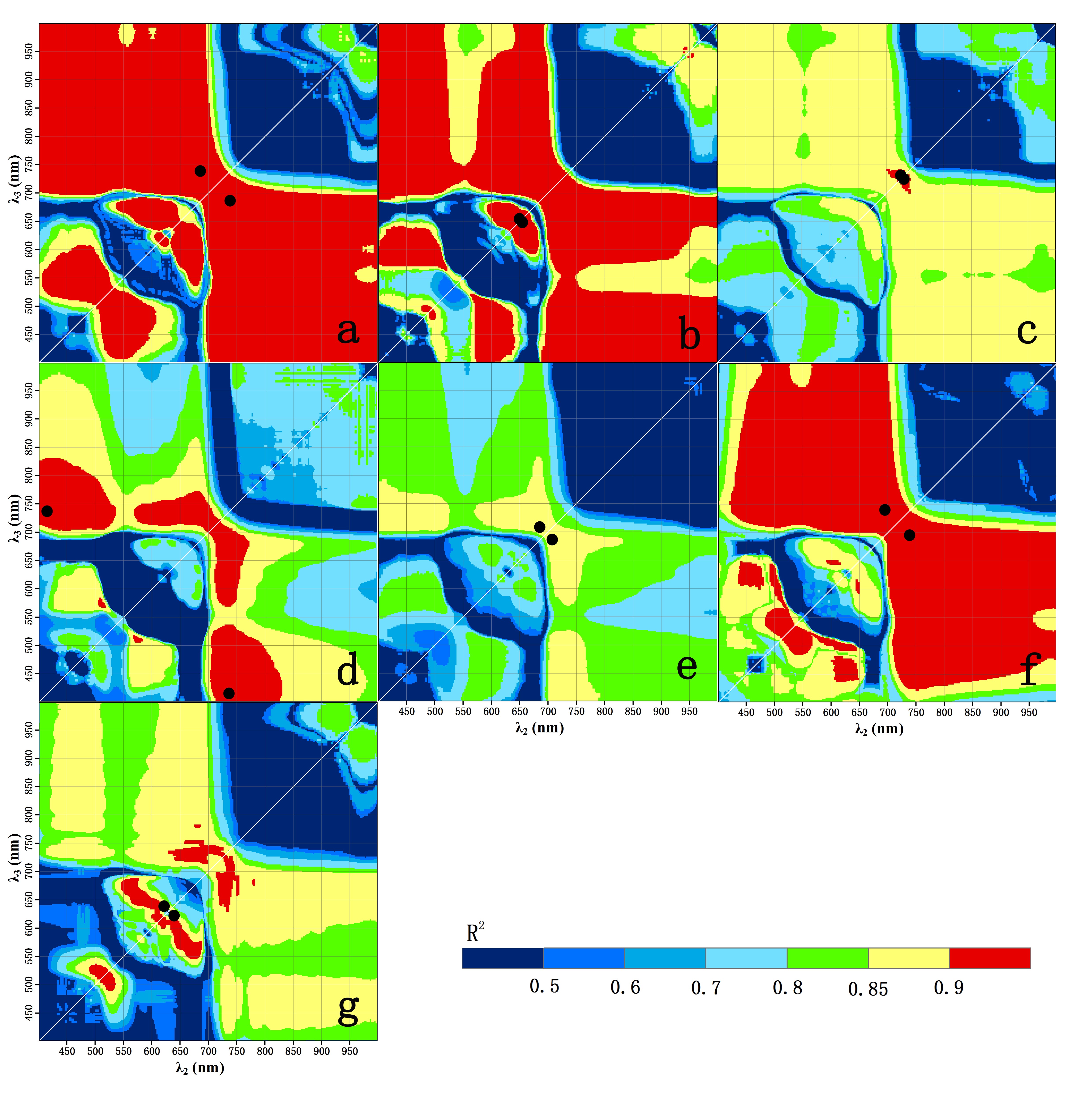

Supplement: Supplementary file 5 — Additional file 5: Figure S4. Contour maps for R2 between LCC and the MDATT index with the combination of λ2 and λ3 derived from the abaxial leaf reflectance for each plant species (the dots and letters represent the same items as in Figure S1). [file 13007_2018_281_MOESM5_ESM.jpg]

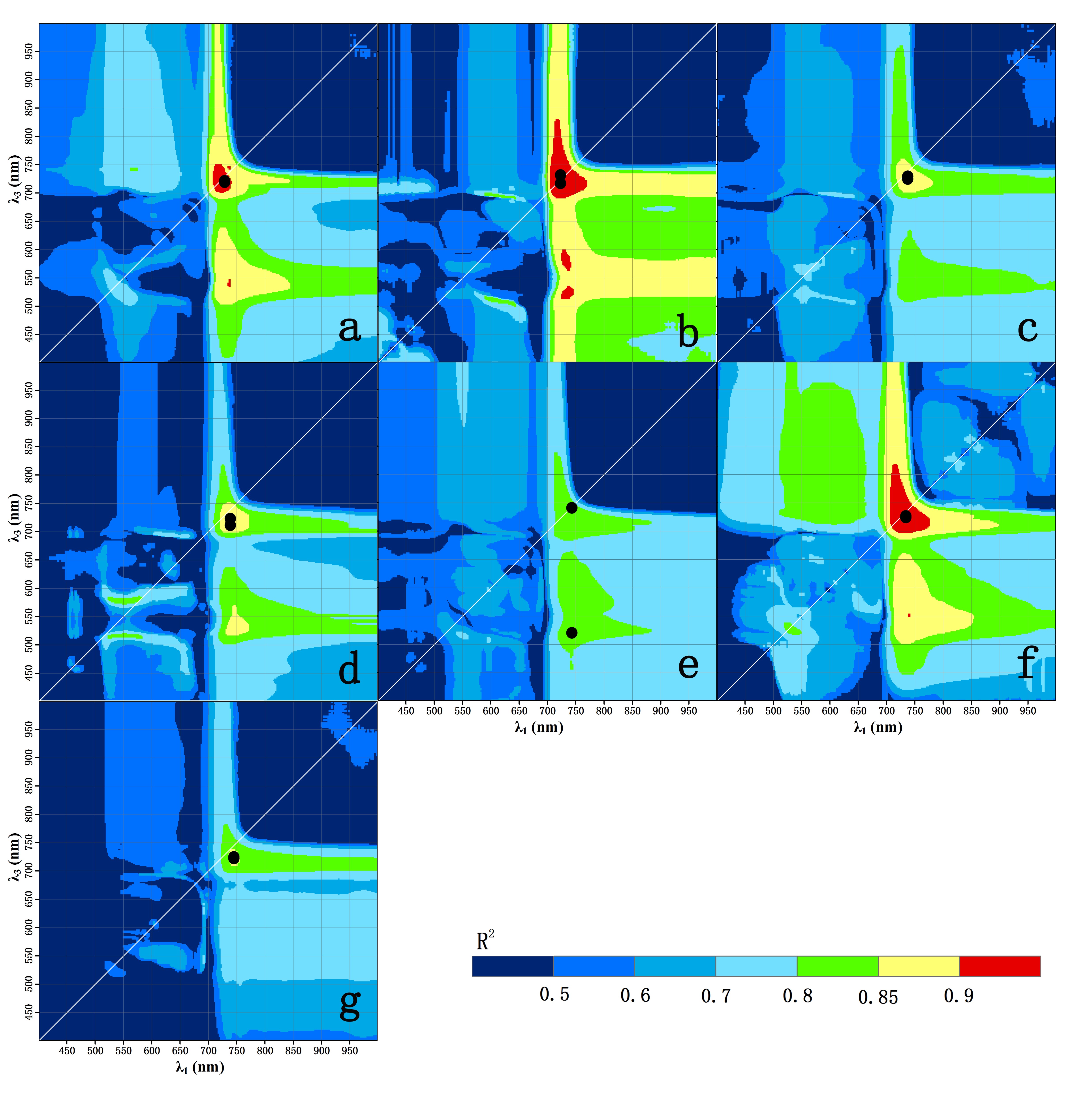

Supplement: Supplementary file 6 — Additional file 6: Figure S5. Contour maps for R2 between LCC and the MDATT index with the combination of λ2 and λ3 derived from the adaxial and abaxial leaf reflectance for each plant species (the dots and letters represent the same items as in Figure S1). [file 13007_2018_281_MOESM6_ESM.jpg]

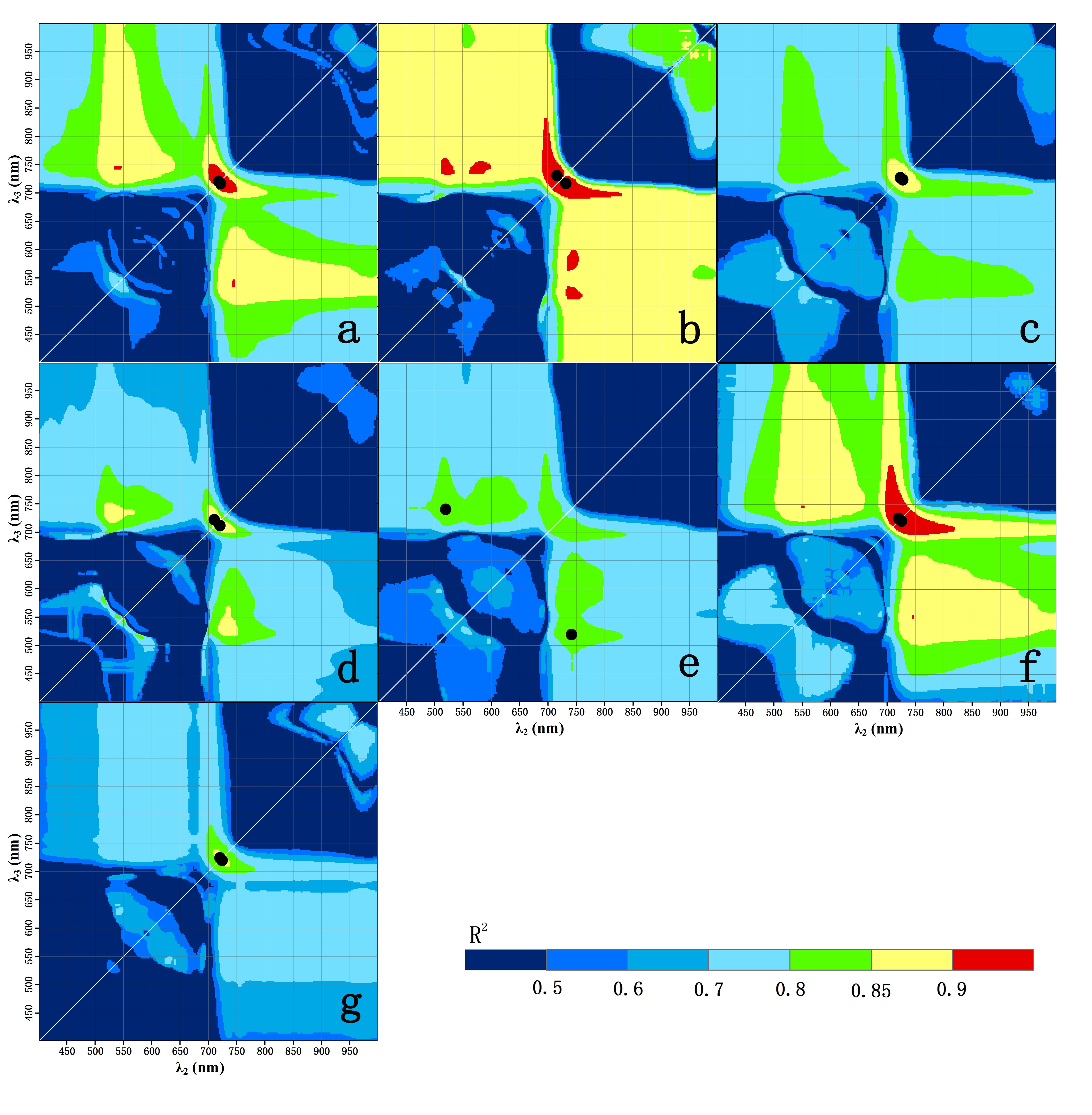

Supplement: Supplementary file 7 — Additional file 7: Figure S6. Contour maps for R2 between LCC and the MDATT index with the combination of λ2 and λ3 derived from the adaxial and abaxial leaf reflectance for each plant species (the dots and letters represent the same items as in Figure S1). [file 13007_2018_281_MOESM7_ESM.jpg]
